# Blueberry Extract Improves Obesity through Regulation of the Gut Microbiota and Bile Acids via Pathways Involving FXR and TGR5

**DOI:** 10.1016/j.isci.2019.08.020

**Published:** 2019-08-16

**Authors:** Jielong Guo, Xue Han, Hongyu Tan, Weidong Huang, Yilin You, Jicheng Zhan

**Affiliations:** 1 College of Food Science and Nutritional Engineering, Beijing Key Laboratory of Viticulture and Enology, China Agricultural University, Beijing 100083, China; 2 Xinghua Industrial Research Centre for Food Science and Human Health, China Agricultural University, Xinghua 225700, Jiangsu, China

**Keywords:** Nutrition, Obesity Medicine, Microbiome

## Abstract

The metabolic improvement effect of blueberries has long been recognized, although its precise mechanism(s) remains obscure. Here, we show that phenolic blueberry extract (BE) treatment improved diet- and genetically induced metabolic syndromes, which were linked to increased energy expenditure in brown adipose tissue (BAT) and improved lipid metabolism in the liver via pathways involving the bile acid (BA) receptors TGR5 and FXR. These observations were strongly correlated with the regulation of BAs (e.g., a decrease in the FXR inhibitors TαMCA and TβMCA) and the gut microbiota (GM) (e.g., an expansion of *Bifidobacteria* and *Lactobacillus*), because antibiotic treatment completely blunted the regulation of the GM and BAs and the metabolic effects of BE. We also observed similar results in *db/db* mice. Furthermore, treating mouse primary cells derived from the liver and BAT with the combinations of BAs mimicking the *in vivo* alterations upon BE treatment mirrored the *in vivo* observations in mice.

## Highlights

• Blueberry extract (BE) treatment improved obesity-related metabolic syndromes in mice
• BE treatment regulated the plasma bile acid (BA) pool through gut microbiota
• BE treatment decreased serum-conjugated BA contents and increased free secondary BAs
• BE treatment activated BA receptors TGR5 and FXR in brown adipose tissue and liver

## Graphical Abstract

**Figure:**
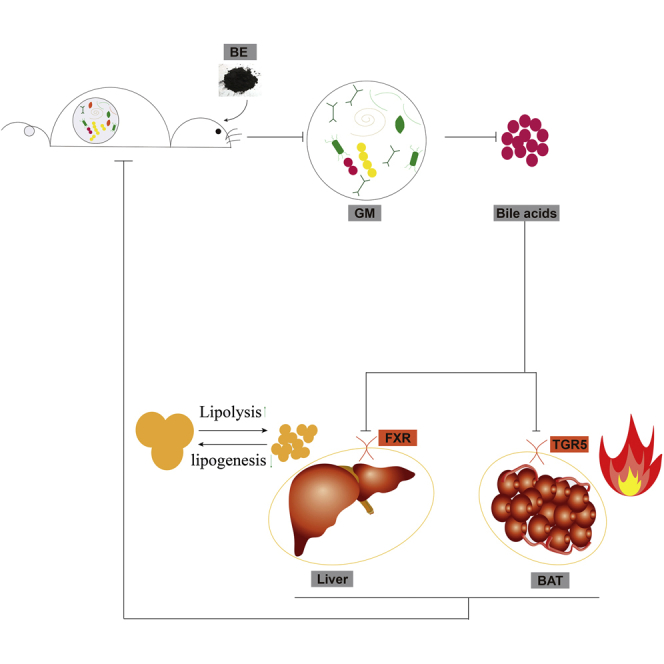

## Introduction

The antimetabolic disease function of plant polyphenols (PPs) has long been noted by researchers studying the “health secrets” of people following Mediterranean diet, which contains abundant PPs (Kastorini et al., 2011). Numerous animal and human studies have demonstrated that, although controversial, PPs from fruits, vegetables, and wines can improve diet-induced obesity and its related metabolic syndromes, including inflammation and insulin resistance (IR) (Han et al., 2018, Tresserra-Rimbau et al., 2015). Certain mechanisms underlying these phenotypes have been studied, such as the inhibition of α-glucosidase, enhancement of pancreatic β-cell function, and regulation of liver function (Hanhineva et al., 2010). However, the reasons for these effects are unclear, as most natural PPs cannot be absorbed directly by mammals, and the plasma concentration of natural PPs or their metabolites is low, making it difficult to fulfill their physiological function (Kay et al., 2017).

Recently, the regulation of the gut microbiota (GM) by PPs has received more attention for its potential to improve metabolism. The GM of mammals has fundamental impacts on the host's health in many aspects, such as immunity, psychology, behavior and, especially, energy metabolism (Turnbaugh et al., 2006, Bäckhed et al., 2004). The GM plays a significant role in regulating the body weight (BW) of hosts (Ridaura et al., 2013). Metabolites of the GM are important in regulating the host's metabolism, and, among others, bile acids (BAs) play a key role (Swann et al., 2011). BAs are synthesized from cholesterol and conjugated to taurine (mouse) or glycine (human) in the liver before being released into the intestine, where they meet microbes. The GM can convert conjugated primary BAs to free BAs via bile salt hydrolase (BSH) and then further to secondary BAs through dehydroxylation and dehydrogenation (Just et al., 2018). BAs can modulate energy homeostasis through G protein-coupled bile acid receptor 1 (GPBAR1, also known as TGR5) and farnesoid X receptor (FXR, also known as BAR). Supplementation with cholic acid (CA) prevented diet-induced obesity and its related metabolic syndromes in mice via the activation of TGR5 (Watanabe et al., 2006).

Blueberries (*Vaccinium spp.*) are rich in PPs (mainly anthocyanins) and known for their antioxidant and cardiovascular protective function (Neto, 2010). It has been shown that blueberries (or their phenolic extract) were able to ameliorate high-fat diet (HFD)-induced obesity and related metabolic syndromes such as hyperglycemia and IR and to regulate the GM composition (Stull et al., 2010, Defuria et al., 2009). However, although a correlation between the regulation of the GM and an improvement in metabolism has been observed, neither has the causal link of this relationship nor has the mechanism of this connection been studied yet (Lee et al., 2018). To investigate the correlation between blueberry PPs, the GM, and metabolism, we performed four independent experiments (three mouse studies and one *in vitro* study, more details seen in Transparent Methods-Animals and Cell Separation and Culture) employing both mouse and mouse primary cells. The results of this study have implications for understanding the mechanism(s) of the effects of dietary PPs on health and the relationship between the GM and host metabolism.

## Results

### Blueberry Extract Alleviates Obesity and Liver Steatosis in Diet-Induced Obese Mice

We obtained 2.81 ± 0.23 g extract, including 79.62% phenolic compounds, from 100 g fresh blueberry fruit. The hexosides of delphinidin, malvidin, petunidin, cyanidin, myricetin, and quercetin as well as 5-caffeoylquinic acid, vanillic acid, and proanthocyanidins were identified as the most abundant components of the blueberry extract (BE) (Table S1).

**Figure 1:**
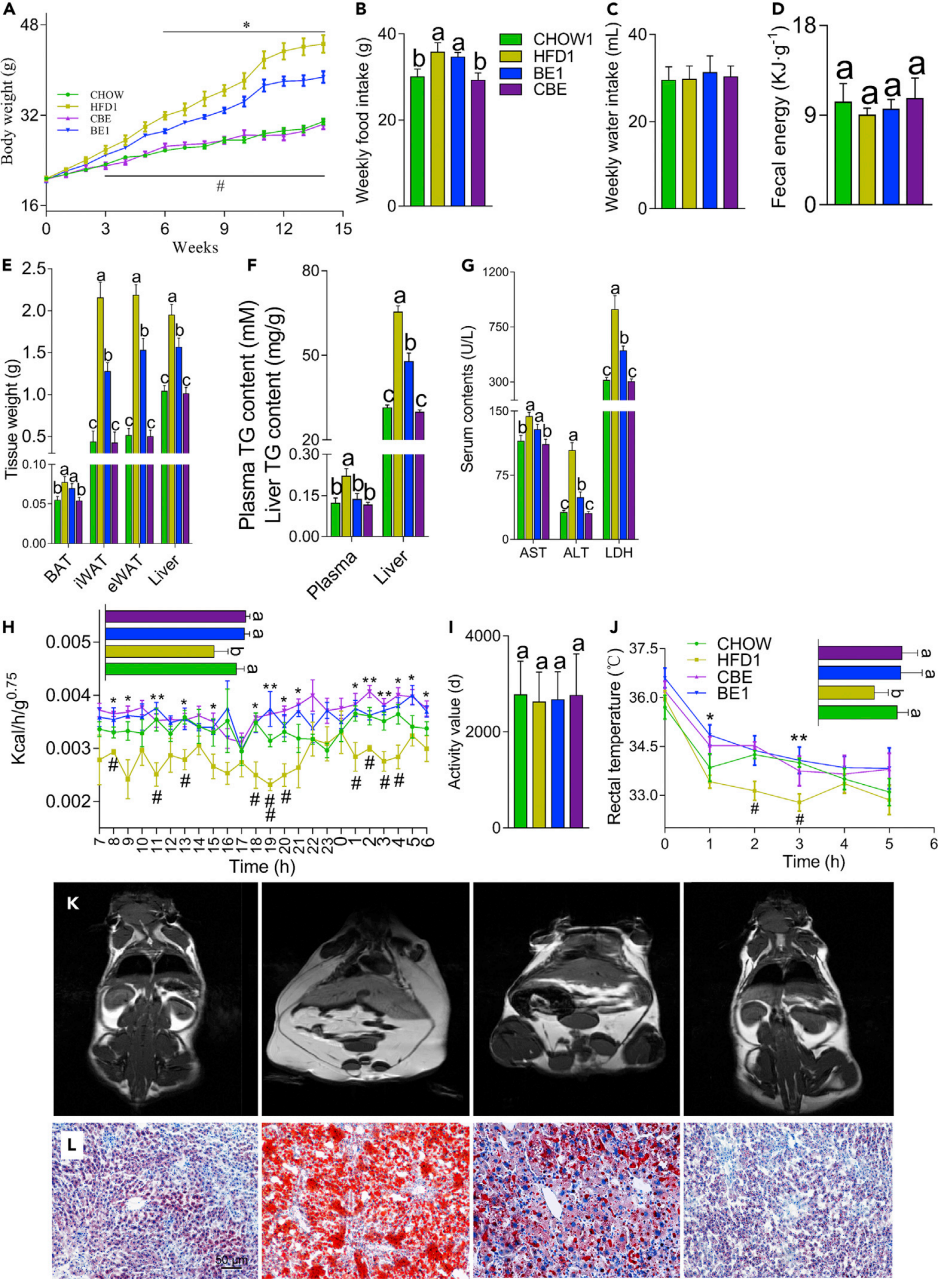
BE Treatment Alleviated Obesity, Reduced Liver Steatosis, and Increased Energy Expenditure (A) The body weight change of mice throughout the experiment in study 1. (B–D) The weekly food intake (B), water intake (C), and fecal energy (D) of mice in study 1. (E) Tissue weights. (F) The TG concentration in the plasma and liver. (G) Plasma concentrations of AST, ALT, and LDH, respectively. (H and I) BE treatment increased the energy expenditure of mice fed an HFD (H), which was not related to physical activity (I). (J) The alteration in rectal temperature following cold stimulation and the relative area under the curve (AUC). (K) Magnetic resonance imaging graphs of the CHOW1, HFD1 BE1, and CBE groups (from left to right). The white areas represent lipids, n = 8. (L) Representative oil red O staining of the livers of mice from the CHOW1, HFD1 BE1, and CBE groups (from left to right), n = 8. For all figures, *p *<* 0.05 and **p *<* 0.01 for BE1 versus HFD1, ^*#*^p *< 0.05* and ^*##*^p *<* 0.01 for CHOW1 versus HFD1 and different letters indicate a significant difference between columns, p < 0.05.

**Figure 2:**
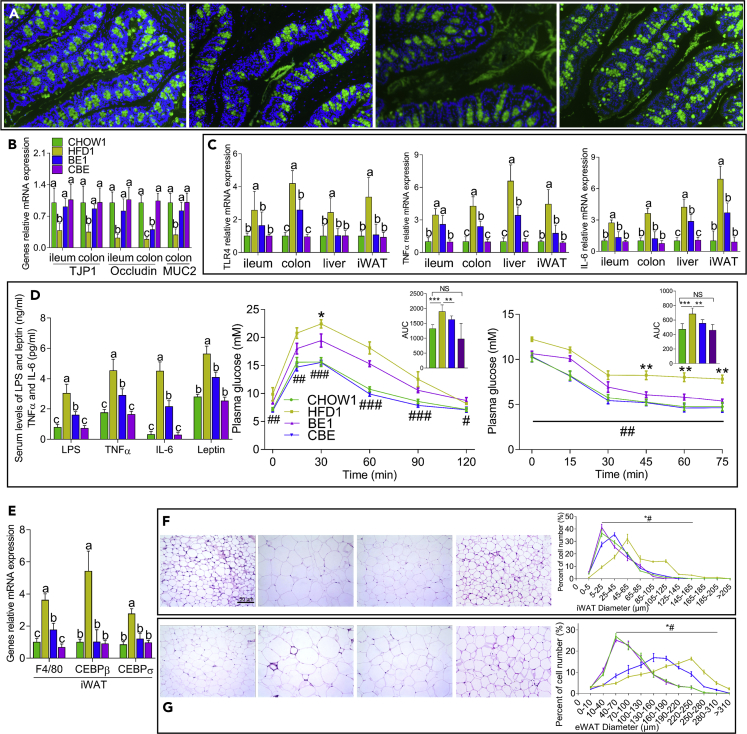
BE Treatment Ameliorated Systemic Inflammation and Improved Glucose Metabolism (A) Fluorescence microscope analysis of colonic MUC2 in mice from the CHOW1, HFD1, BE1, and CBE groups (from left to right). MUC2, green; DNA, blue; n = 10. (B and C) The relative mRNA expression of genes related to intestinal permeability (B) and inflammation (C). (D) The serum contents of inflammatory factors and changes in blood glucose concentrations. *∗*p *<* 0.05, *∗∗*p *<* 0.01 and ∗∗∗p < 0.001 for BE1 versus HFD1 and ^*#*^p *<* 0.05, ^*##*^p *<* 0.01, and ^*###*^p *<* 0.001 for CHOW1 versus HFD1. (E) The relative mRNA expression of genes in iWAT. (F and G) Representative pictures showing the hematoxylin and eosin staining of iWAT (F) and eWAT (G) in CHOW1, HFD1, BE1, and CBE mice (from left to right) and the percentage of the cells with different diameters, *∗*p *<* 0.05 for BE1 versus HFD1 and ^*#*^p *<* 0.05 for CHOW1 versus HFD1. For all pictures, different letters indicate a significant difference between columns, p *<* 0.05.

For mouse study 1- and 3-week old C57BL/6 male mice were randomly assigned to four groups after adapting for 1 week as follows: (1) a CHOW1 group fed a standard chow diet (3.85 kcal/g, 10% energy from fat), (2) an HFD1 group fed an HFD (4.73 kcal/g, 60% energy from fat), (3) a CBE group (5 gL^−1^ BE in drinking water) fed a standard chow diet, and (4) a BE1 group (0.5% (m/v) BE in drinking water) fed an HFD. BE treatment significantly reduced diet-induced weight gain from the sixth week of the study until the end of the study, and these findings were not related to energy intake or excretion (Figures 1A, 1B, and 1D). Moreover, the water consumption was also not influenced by BE treatment (Figure 1C). BE-treated mice had reduced body fat, especially in the inguinal white adipose tissue (iWAT), epididymal white adipose tissue (eWAT), and liver (Figures 1E, 1K, 1L, 2F, and 2G). Liver steatosis and damage induced by an HFD were significantly ameliorated through BE administration, as indicated by decreased hepatic and plasma concentrations of triacylglycerol (TG) (Figure 1F) and a reduction in plasma lactate dehydrogenase, alanine transaminase, and aspartate aminotransferase content (Figure 1G). BE-treated mice showed an increased energy expenditure (Figure 1H), which was not related to physical activity (Figure 1I); consistent with this, the core body temperature of BE-treated mice was higher than that of the vehicle-treated HFD-fed mice (Figure 1J). Notably, BE treatment had no significant influence on the metabolism of mice fed a chow diet, as shown by parameters such as BW, tissue weight, fat deposition, and plasma biochemical parameters compared with those of chow-fed control mice (Figure 1). Taken together, these results showed that BE administration reduced HFD-induced weight gain and adiposity (partially) by increasing energy expenditure and thermogenesis.

### BE Treatment Ameliorates Increased Intestinal Permeability and Systemic Inflammation Triggered by an HFD and Improves Glucose Metabolism

BE treatment alleviated the increased permeability of the colon and distal ileum triggered by an HFD, as indicated by the increased MUC2 levels in the colon and increased mRNA expression of occludin and tight junction protein 1 (TJP1, also known as ZO-1) both in the ileum and colon compared with their expression in mice in the HFD1 group (Figures 2A and 2B), which was accompanied by a reduction in the mRNA levels of genes related to inflammation, including toll-like receptor 4 (TLR4), interleukin 6 (IL-6), and tumor necrosis factor alpha (TNF-α) (Figure 2C). We also found an improvement in inflammation in white adipose tissue (WAT) and the liver in BE-treated mice, as indicated by the reduced mRNA expression of TLR4, IL-6, and TNF-α and the decreased adipocyte size in the WAT (Figures 2C, 2F and 2G). Consistent with these results, the plasma concentrations of lipopolysaccharides (LPS), IL-6, and TNF-α were also decreased in BE-treated mice compared with those in vehicle-treated HFD-fed mice (Figure 2D).

Glucose metabolism is closely related to systemic inflammation (Gregor and Hotamisligil, 2011). As expected, BE-treated mice showed profoundly improved glucose intolerance and insulin sensitivity (Figure 2D). Notably, leptin, a key modulator of energy homeostasis that is usually irregularly high in obese individuals (Friedman and Halaas, 1998), was lower in the plasma of mice in the BE1 group than in mice in the HFD1 group, and, consistent with this, BE-treated mice showed reduced mRNA expression of CCAAT enhancer-binding proteins β and σ (CEBPβ and CEBPσ) in the iWAT (Figure 2E). Similar to the previous observations, systemic inflammation and glucose metabolism in BE-treated chow-fed mice resembled those of vehicle-treated chow-fed mice. Collectively, these results showed that BE administration enhanced the expression of genes related with intestinal barrier integrity, decreased systemic inflammation, and improved glucose metabolism.

### BE Improves the GM Disturbed by HFD

**Figure 3:**
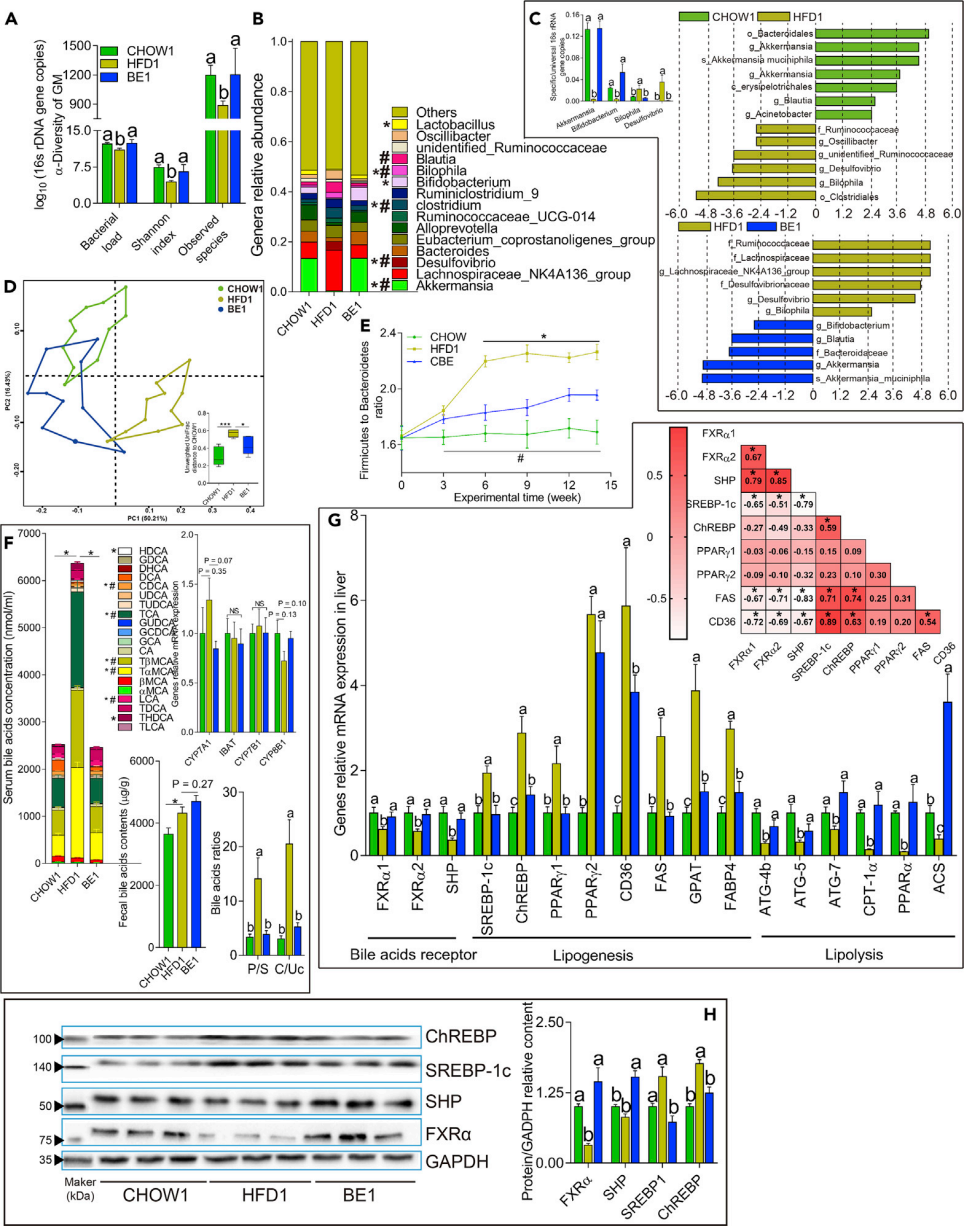
BE Administration Ameliorated the HFD-Induced Disturbance of the GM (A) BE increased the α-diversity of the GM and the total bacterial load (based on 16S rRNA gene copies). (B) Relative abundance of the top genera. (C) The abundance of bacterial genera (left) identified by LEfSe analysis (right) in mice from the HFD1 and BE1 groups. (D) Principal coordinate analysis (PCoA) of the GM based on the unweighted UniFrac distance. (E) The alteration in the Firmicutes to Bacteroidetes ratio based on qPCR. (F) BE administration changed the plasma BA pool size and composition (upper left) and the ratios of primary to secondary and conjugated to unconjugated BAs (bottom right), which was not related to the reabsorption or synthesis (upper right and bottom left) of BAs. P/S, primary BAs/secondary BAs; C/Uc, conjugated BAs/unconjugated BAs. (G) The mRNA expression of genes related to lipid metabolism in the liver (bottom) and the correlation matrix between the mRNA expression levels of key genes controlling lipogenic and BA receptors (upper); r values and significance were according to Spearman's rank correlation test (*∗*p *<* 0.05 if 0.362 < r < 0.467; *∗*p *<* 0.01 if 0.467 < r < 0.580; *∗*p *<* 0.001 if r > 0.580). (H) Representative western blots showing the expression of proteins in the livers of mice from the CHOW1, HFD1, and BE1 groups (n = 3).

Based on the above results, we concluded that BE administration had a minimal influence on the metabolism of mice fed a chow diet; therefore, we excluded the mice in the CBE group in the following analysis and experiments. Fourteen weeks of treatment with an HFD decreased the richness of the GM and resulted in an abnormal Bacteroidetes to Fimicutes ratio in the HFD1 mice, which were completely restored through BE administration (Figures 3A and 3E). Intergroup analysis based on the unweighted UniFrac distance showed that BE-treated mice were more similar to mice fed a chow diet (Figure 3D) than vehicle-treated HFD-fed mice. Principal coordinate analysis based on the unweighted UniFrac distance also revealed that BE-treated mice were clustered apart from vehicle-treated HFD-fed mice and partially overlapped with mice fed a chow diet (Figure 3D).

We employed the linear discriminant analysis effect size (LEfSe) approach to determine the bacterial taxa notably influenced by BE treatment. Consistent with the above results, taxa belonging to the phyla Bacteroidetes and Firmicutes, such as Ruminococcaceae and Bacteroidaceae, were the main features discriminating the GM of BE-treated mice from that of vehicle-treated HFD-fed mice (Figure 3C). In addition, the expansion of *Akkermansia* and *Bifidobacterium* along with a significant decrease in the *Desulfovibrio* and *Bilophila* genera was observed in mice in the BE1 group (Figure 3C). Moreover, the abundance of the significant taxa identified through LEfSe analysis was considerably high, as determined through sequencing and qPCR (Figures 3B and 3C). Collectively, these results demonstrated a substantial alteration and improvement in the GM through BE administration.

### BE Changes the Plasma Bile Acid Pool, Promotes the Activation of BAT and the Browning of WAT, and Improves Lipid Metabolism in the Liver and Adipose Tissue

As BA metabolism is closely connected with the GM, and they are important regulators of energy homeostasis (Swann et al., 2011), we sought to determine the influence of BE administration on the BA pool and metabolism. BE administration substantially decreased the augmented plasma BA size induced by an HFD, and conjugated BAs, including tauro-α-muricholic acid (TαMCA), TβMCA, and taurocholic acid (TCA), were the most reduced BAs, among others (Figure 3F). Among other highly abundant BAs, lithocholic acid (LCA) and chenodeoxycholic acid (CDCA) were increased; CA, deoxycholic acid (DCA), ursodeoxycholic acid, and tauroursodeoxycholic acid (TUDCA) were not influenced; and hyodeoxycholic acid (HDCA) and tauro-HDCA were decreased in BE-treated mice compared with their levels in mice in the HFD1 group (Figure 3F). Furthermore, the ratios of primary to secondary as well as conjugated to unconjugated BAs were decreased following BE treatment (Figure 3F). The *Bifidobacterium* and *Bacteroides* genera, known to produce BSH (Gerard, 2013), were strongly positively correlated with some unconjugated BAs (e.g., LCA and CA) in the plasma and negatively related to taurine-conjugated BAs including TαMCA, TβMCA, and TCA (Figure S1C). The fecal BA level was slightly (p = 0.27) increased, whereas the mRNA expression of ileal bile acid transporter (IBAT, also known as SLC10A2 and ABST) was not influenced by BE treatment (Figure 3F), suggesting that the increase in secondary BAs may contribute to the reduced intestinal reabsorption of BAs (Sayin et al., 2013). Collectively, these results showed that BE administration altered the plasma BA pool and the GM and that there was a strong correlation between the alteration of BA pool and GM.

FXR, an important nuclear BA receptor, controls the expression of various genes related to lipid metabolism, such as sterol-regulatory element binding proteins-1c (SREBP-1c) and carbohydrate-responsive element-binding protein (ChREBP), in the liver (Molinaro et al., 2018). As BE administration sharply decreased the plasma levels of TαMCA and TβMCA, FXR antagonists (Sayin et al., 2013), we assessed the influence of their reduced levels on FXR-dependent lipid metabolism. As expected, both the mRNA and protein levels of FXRα and small heterodimer partner (SHP) were significantly elevated, along with the suppression of SREBP-1c and ChREBP, in BE-treated mice compared with those in mice in the HFD1 group (Figures 3G and 3H). The mRNA expression levels of genes downstream of SREBP-1c and ChREBP, including proliferator-activated receptor γ1 (PPARγ1), PPARγ2, fatty acid translocase (CD36), fatty acid synthase (FAS), glycerol-3-phosphate acyltransferase (GPAT), and fatty acid-binding protein 4 (FABP4), were all decreased in the livers of BE-treated mice compared with those of vehicle-treated HFD-fed mice (Figure 3G). Notably, there were strong correlations between the mRNA expression levels of FXRα, SHP, SREBP-1c, ChREBP, FAS, and CD36 (Figure 3G). In contrast, the mRNA levels of proteins related to lipolysis, including autophagy-related 4B cysteine peptidase (ATG4b), autophagy-related protein 5 (ATG5), ATG7, carnitine palmitoyltransferase (CPT-I), PPARα, and acetyl-coenzyme A synthetase (ACS), were increased in the livers of BE-treated mice compared with their levels in mice in the HFD1 group (Figure 3G). The expression of proteins related to lipid metabolism in WAT and brown adipose tissue (BAT) mirrored the results in the liver, except that FXRα and SHP were not influenced by BE (Figures S1A and 1B). Moreover, BE treatment reduced the mRNA expression of cytochrome P450 7A1 (CYP7A1), the key protein controlling BA synthesis, in the liver (Figure 3F), which was consistent with the reduced plasma BA pool size.

**Figure 4:**
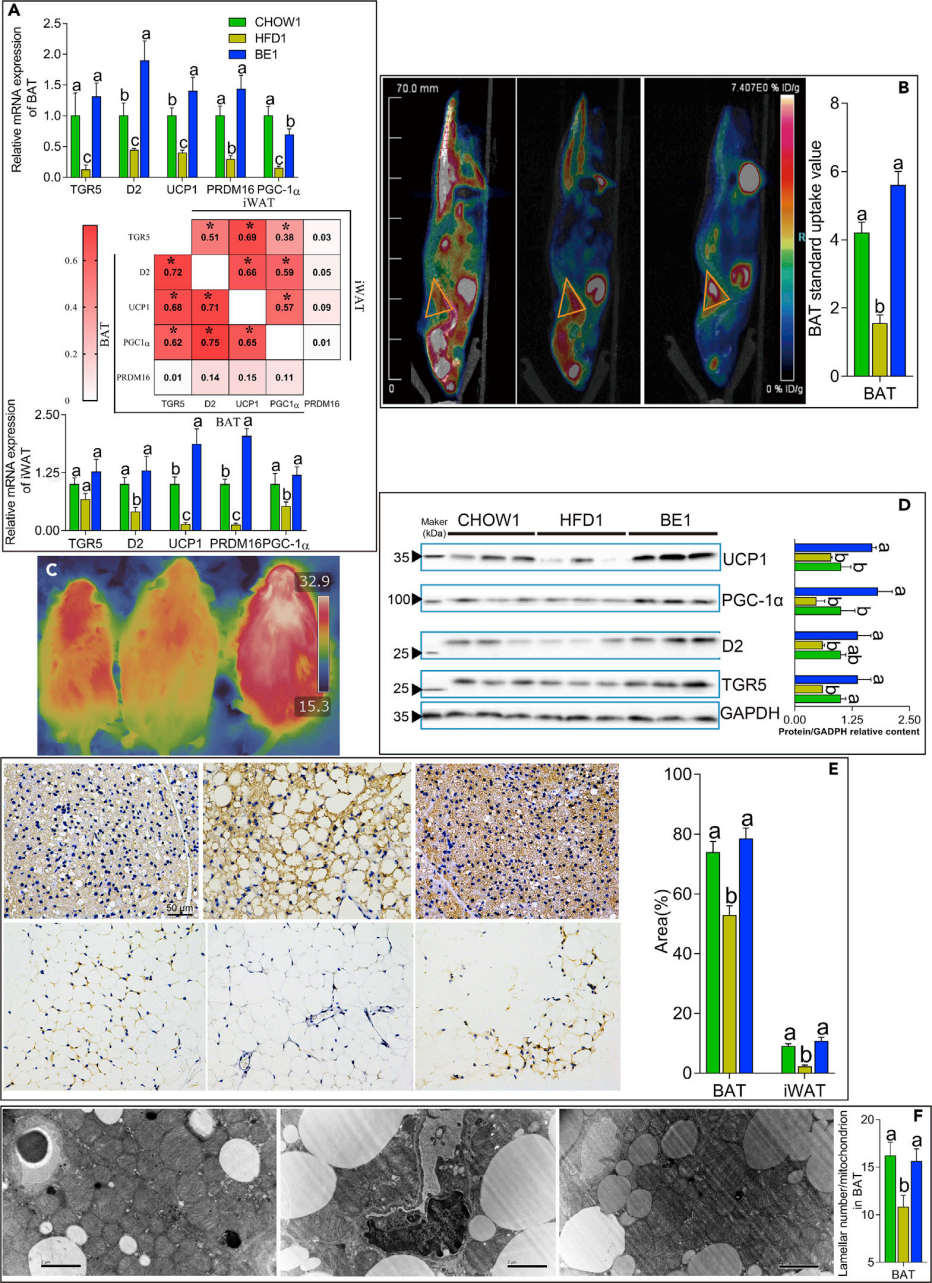
BE Administration Enhanced the Function of BAT and the Browning of iWAT (A) The relative mRNA expression of genes related to the activation of BAT (up) and the browning of iWAT (down); correlation matrix between the mRNA expression of genes in BAT and iWAT (middle); r values and significance were according to Spearman's rank correlation test (*∗*p *<* 0.05 if 0.362 < r < 0.467; *∗*p *<* 0.01 if 0.467 < r < 0.580; *∗*p *<* 0.001 if r > 0.580). (B) Representative PET-CT scan of mice after mild cold stimulation. Yellow triangles indicate the anatomical site of the interscapular BAT, n = 5. (C) Representative infrared thermal images, n = 8. (D) Representative western blots showing the expression of proteins in BAT sections of mice from the CHOW1, HFD1, and BE1 groups (n = 3). (E) Representative immunohistochemistry to detect UCP1 (brown stain) in BAT (upper left) and iWAT (bottom left) sections and the area under the curve AUC (right), n = 10. (F) Representative transmission electronic microscopy images from BAT showing that BE treatment increased the lamellar cristae number in the mitochondria. Scale bar, 2 μm, original magnification 12,000×, n = 8. For (B, C, E, and F), the images represent mice from the CHOW1, HFD1, and BE1 groups from left to right. For all figures, different letters indicate a significant difference between columns, p *<* 0.05.

We then sought to determine the effect of BE on the activation of TGR5 in BAT. BE-treated mice showed elevated levels of the membrane BA receptor TGR5 and its downstream gene, deiodinase 2 (D2), in BAT (Figures 4A and 4D). Consistent with this, both the mRNA and protein expression of genes related to nonshivering thermogenesis (NST), including uncoupling protein-1 (UCP1), PR domain-containing 16 (PRDM16), and peroxisome proliferator-activated receptor gamma coactivator 1-alpha (PGC-1α), were increased in the BAT of BE-treated mice compared with their expression in vehicle-treated HFD-fed mice (Figures 4A, 4D, and 4E). There were strong positive correlations between the mRNA expression levels of TGR5, D2, UCP1, PGC-1α, and PRDM16 (Figure 4A). Consistent with these observations, we found significantly increased BAT functioning following cold stimulation through positron emission tomography-computed tomography (PET-CT) (Figure 4B) and an increased intrascapular temperature in BE-treated mice (Figure 4C), suggesting more powerful NST in the BAT of BE-treated mice. We also found more lamellar cristae in the mitochondria in the BAT of BE-treated mice than in the BAT of vehicle-treated HFD-fed mice (Figure 4F). In addition, our results also revealed a significant increase in the key proteins involved in the browning of adipocytes in iWAT, including D2, UCP1, PGC-1α, and PRDM16, although the mRNA expression of TGR5 (p = 0.07) was not significantly influenced by BE administration (Figures 4A and 4E). Collectively, these results suggested that BE administration promoted NST in BAT and the browning of iWAT through the BA membrane receptor TGR5, thereby increasing the energy expenditure and (in part) contributing to the anti-obesity effect of BE.

### BE Improves Obesity and Its Related Metabolic Syndromes in *db/db* Mice by the Alteration of the GM and BAs

**Figure 5:**
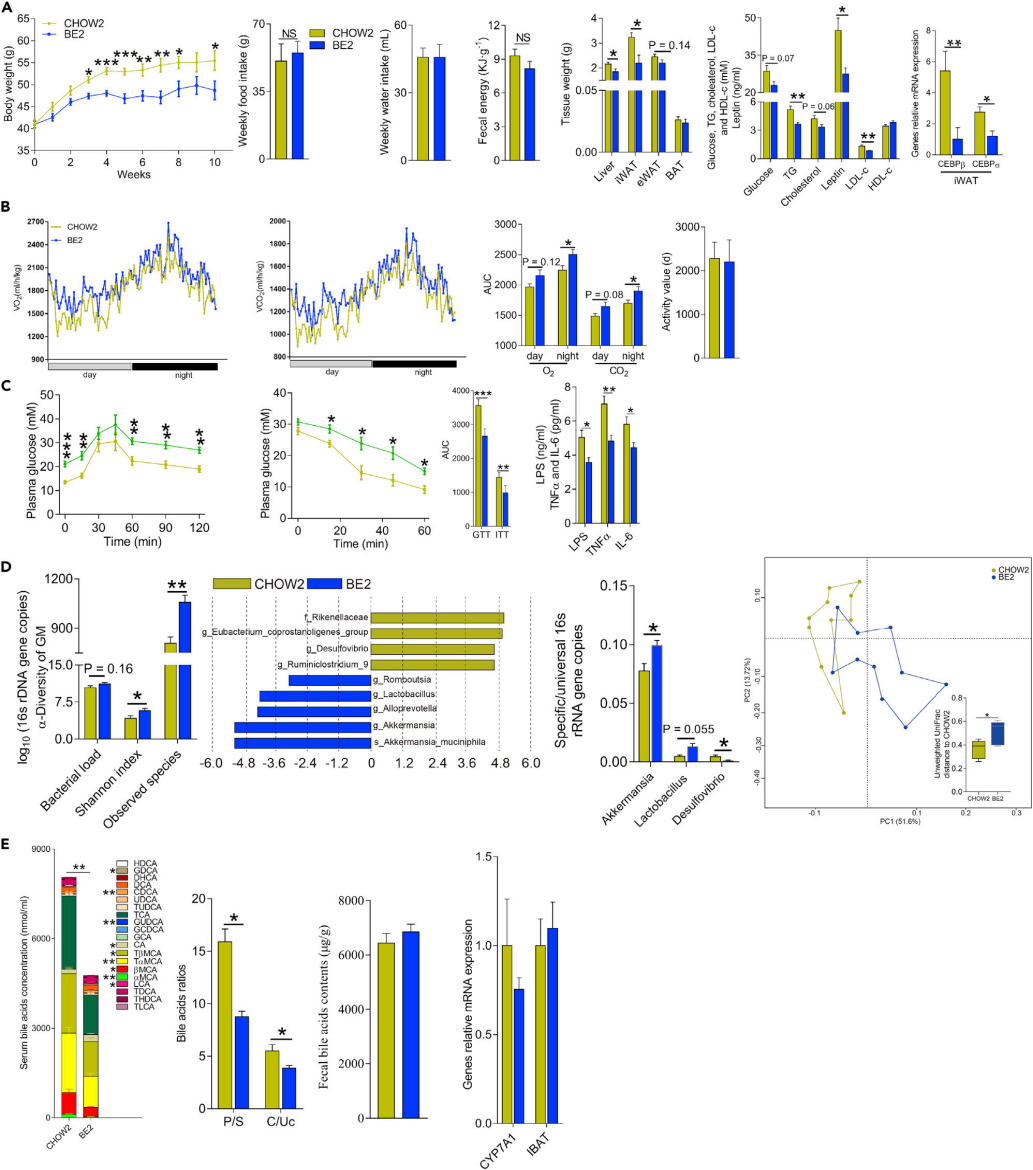
BE Treatment Improved Metabolic Syndromes by the Regulation of the BA Pool and the GM in *db/db* Mice (A) The body weight (upper), food intake, water intake, fecal energy, tissue weights, plasma parameters, and gene expression (from left to right) of mice in study 2. (B) Energy expenditure (left and middle) and physical activity (right) of mice. (C) BE administration improved systemic inflammation (right), glucose intolerance (left and middle), and insulin resistance (middle). (D) The α-diversity (left) and PCoA analysis of GM (right). The richness of certain bacterial genera identified by LEfSe (middle). (E) Plasma BA composition (left and second from left), fecal BA contents (third from left), and the relative mRNA expression (right). For all figures, *∗*p *<* 0.05, *∗∗*p *<* 0.01, and *∗∗∗*p *<* 0.001.

In mouse study 2, male C57BL/KsJ *db/db* mice purchased at 21 days of age were randomly assigned to two groups (n = 10) as follows: (1) a CHOW2 group fed a chow diet and (2) a BE2 group fed a chow diet with 5 gL^-1^ BE in drinking water. BE administration decreased genetically induced weight gain with no significant influence on the water consumption, energy intake, and energy in the feces, which was mainly the result of the decreased tissue weight of the liver, iWAT, and eWAT (Figure 5A). Liver steatosis, systemic inflammation, fat deposition and glucose metabolism were all improved by BE treatment (Figures 5A, 5C, S2, and S3A). BE-treated mice also had reduced serum TG, cholesterol, and leptin levels compared with mice in the CHOW2 group (Figure 5A). The improvement in adiposity was related to increased energy expenditure and an improvement in lipid metabolism (Figures 5B and S3A). BE-treated mice showed elevated mRNA expression of genes related to NST in iWAT and BAT and enhanced BAT functioning (Figures 5B–5E). There were strong correlations of the mRNA expression levels between TGR5, D2, PGC-1α, and PRDM16 and between FXR, SHP, SREBP-1c and ChREBP, suggesting that the regulation of lipid metabolism and enhancement of NST is closely linked to the activation of BA receptors FXR and TGR5 (Figures S3A and S3D).

BE administration increased the diversity of the GM, decreased the abundance of Proteobacteria and the ratio of Firmicutes to Bacteroidetes, and caused a significant difference in the GM of BE-treated mice compared with that of mice in the CHOW2 group, as based on the UniFrac distance (Figure 5D). Similar to the results of study 1, BE-treated mice had a lower level of plasma BAs, especially taurine-conjugated primary BAs (mainly TαMCA and TβMCA), and a higher ratio of secondary BAs (mainly LCA and CA) than control mice (Figure 5E). We did not observe differences in the fecal BA content or the mRNA expression in the IBAT between the mice in the two groups (Figure 5E), which was slightly different from the results of study 1. Moreover, there was a close correlation between the GM and plasma BA pool (Figure S1D). In summary, consistent with the results of study 1, BE administration improved genetically induced obesity and its related metabolic syndromes by the activation of TGR5 and FXR via the regulation of the GM and the plasma BA pool.

### Antibiotic Treatment Blunts the Improvement in Metabolism by BE

**Figure 6:**
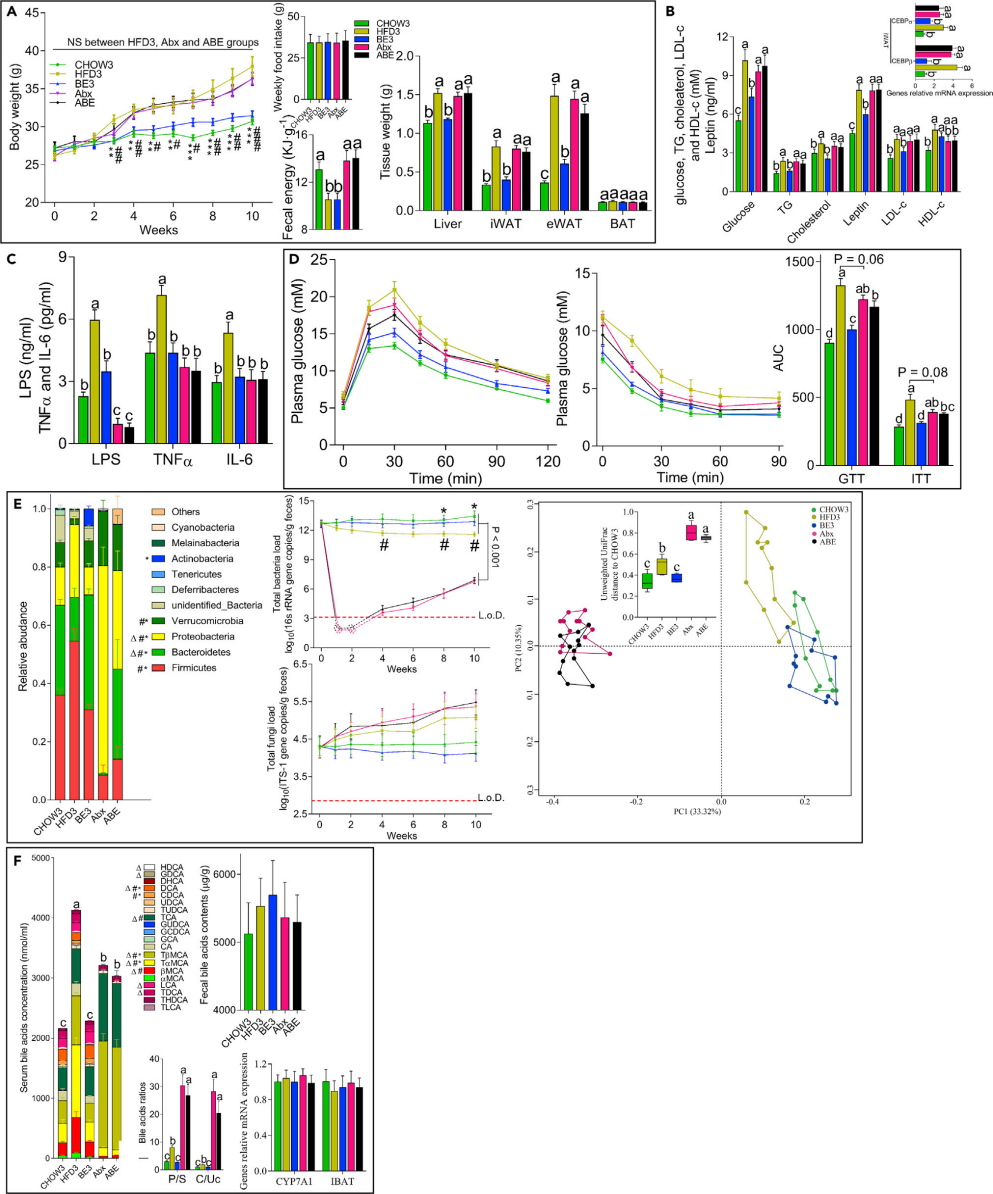
Antibiotic Treatment Blunted the Metabolic Improvement Effect in BE-Treated Mice (A) Antibiotics blunted the anti-adiposis effect of BE (left and right), which was not related to energy intake or excretion (middle). (B–D) There were no significant differences in serum parameters related to metabolic disease (B), systemic inflammation (C), and glucose metabolism (D) between mice in the Abx and ABE groups. (E) Antibiotics substantially changed the composition of the GM (left and right) and decreased the abundance of gut bacteria (upper middle) but had no influence on the fungal richness (bottom middle). *∗*p *<* 0.05 for HFD3 versus CHOW3, ^*#*^p *<* 0.05 for HFD3 versus BE3, and *ΔP < 0.05* for HFD3 versus Abx/ABE. (F) Antibiotic treatment completely changed the plasma BA pool size and composition (left and bottom middle) but had no significant influence on the reabsorption or synthesis of BAs (bottom right) or the fecal concentration of BAs (upper right). *∗*p *<* 0.05 for HFD3 versus CHOW3, ^*#*^p *<* 0.05 for HFD3 versus BE3, and *ΔP <* 0.05, for HFD3 versus Abx/ABE. For all figures, different letters indicate a significant difference between columns, p *<* 0.05.

Mouse study 3 was performed to determine whether there was a causal link between the regulation of the GM, the BA pool, and the improvement in metabolism upon BE administration. In this study, adult (8-week-old) mice were used to minimize the side effects of antibiotics on metabolism as distribution of GM through antibiotics during early life time is strongly linked to various disorders such as obesity, type 1 diabetes, and allergy (Cox and Blaser, 2015, Livanos et al., 2016). Male C57BL/6 mice purchased at 49 days of age were randomly assigned to five groups (n = 10–12) as follows: (1) a CHOW3 group fed a chow diet, (2) an HFD3 group fed an HFD, (3) a BE3 group (5 gL^-1^ BE in drinking water) fed an HFD, (4) an Abx group fed an HFD, and (5) an ABE group (5 gL^-1^ BE in drinking water) fed an HFD. All the mice were gavaged daily with 200 μL PBS containing (for the Abx and ABE groups) 0.5 mg mL^−1^ ampicillin, 0.5 mg mL^−1^ gentamicin, 0.5 mg mL^−1^ metronidazole, 0.5 mg mL^−1^ neomycin, and 0.25 mg mL^−1^ vancomycin or no antibiotics (for the CHOW3, HFD3, and BE3 groups). Consistently, antibiotics had no significant influence on BW (Figure 6A), suggesting that the side effect of antibiotics on the BWs of adult mice was negligible. Antibiotics substantially reduced the gut bacteria to undetected levels during the first two weeks, whereas the abundance of fungi was not significantly influenced throughout the experiment (Figure 6E). Until the end of the experiment, the bacterial richness in antibiotic-treated mice was still quite low (approximately 10^7^·g^−1^ feces), despite the increased tolerance to the antibiotics with time (Figure 6E). The fecal anthocyanin content was significantly higher in antibiotic-treated mice than in BE-treated antibiotic-free mice (Table S1), suggesting the impaired microbial degradation of anthocyanins. We also found that the GM of mice in the Abx and ABE groups was more similar than that of mice in the HFD and BE groups based on the unweighted UniFrac distance (Figure 6E). Antibiotic treatment sharply decreased the abundance of total, secondary, and free BAs compared with those in the antibiotic-free mice (Figure 6F). There was no significant difference in the plasma BA pool size and composition between mice in the Abx and ABE groups (Figure 6F). Taken together, these results suggest that antibiotics substantially blocked the interaction between BE and the GM, thereby destroying the regulation of the BA pool through the GM upon BE administration.

The disturbance of the regulation of the BA pool with antibiotic treatment blunted the activation of TGR5 and FXR through BE administration. There were no differences in the mRNA expression levels of FXR and TGR5 and their downstream genes SHP, SREBP-1c, ChREBP, D2, UCP1, PRDM16, and PGC-1α in the antibiotic- and antibiotic-BE-treated HFD-fed mice (Figure S6). Immunohistochemical analysis further showed that antibiotic-BE-treated mice exhibited similar protein expression of TGR5, D2, UCP1, FXR, SHP, and SREBP-1c as that of antibiotic- and vehicle-treated HFD-fed mice (Figure S7). The observations from PET-CT (data not shown), metabolic cage experiments, and transmission electron microscopy were consistent with these observations (Figure S4). Antibiotic-BE-treated mice had BWs, tissue weights, hepatic fat deposition, and serum TG and cholesterol contents similar to those of antibiotic- and vehicle-treated HFD-fed mice (Figures 6A, 6B, and S5). The improvement in glucose metabolism upon BE administration was partially but not fully counteracted by antibiotics, which may because of the improved inflammation upon antibiotics treatment (Figures 6C and 6D). Collectively, these data suggest that antibiotic treatment damaged the interaction between BE, the GM, and BAs; blunted the activation of TGR5 and FXR; and impaired the metabolic improvement effect of BE.

### DCA and LCA Activate the TGR5 Pathway in Mouse Primary Adipocytes Derived from BAT, whereas TαMCA and TβMCA Inhibit the FXR Pathway in Mouse Primary Hepatocytes in a Dose-Dependent Manner

**Figure 7:**
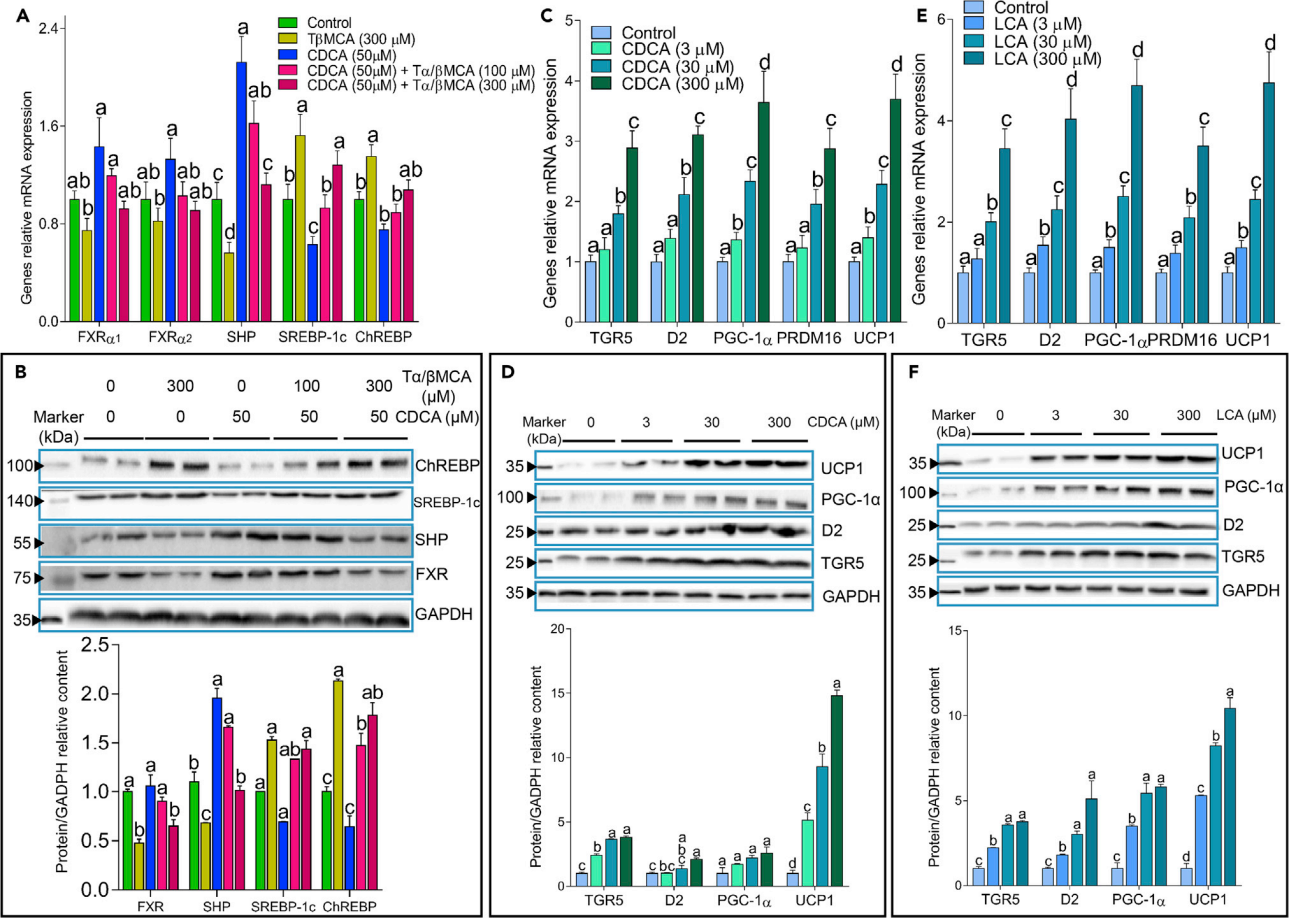
Tα/βMCA Suppressed the FXR Pathway, while CDCA and LCA Enhanced the TGR5 Pathway in Mouse Primary Cells (A and B) Genes relative mRNA expression (A) and western blots (B) in mouse primary hepatocytes showed that CDCA enhanced the mRNA and protein expression of FXR and SHP while suppressing that of SREBP-1c and ChREBP, which was counteracted by Tα/βMCA treatment. (C–F) Genes relative mRNA expression (C and E) and western blots (D and F) in mouse primary adipocytes derived from BAT showed that CDCA (C and D) and LCA (E and F) enhanced the mRNA and protein expression of TGR5 and its downstream genes related to thermogenesis; n = 2. For all images, different letters indicate a significant difference between columns, p < 0.05.

We then sought to determine whether these specific alterations in BAs *in vivo* could be reproduced *in vitro* using mouse primary adipocytes and hepatocytes derived from BAT and the liver, respectively. CDCA and LCA (3 μM) enhanced the expression of TGR5, D2, PGC-1α, and UCP1 in mouse primary adipocytes derived from BAT, and higher doses had a more significant influence (Figures 7C–7F). We next analyzed the effect of high levels of TαMCA and TβMCA on mouse primary hepatocytes with or without the presence of CDCA and found that CDCA increased the levels of FXR and SHP while suppressing the mRNA and protein expression of SREBP-1c and ChREBP (Figures 7A and 7B). The supplementation of TαMCA and TβMCA counteracted the effect of CDCA, and higher levels of TαMCA and TβMCA had a stronger effect, suggesting that TαMCA and TβMCA suppressed the activity of FXR and SHP in a dose-dependent manner (Figures 7A and 7B). Collectively, these results showed that the regulation of the plasma BA pool, mainly the reduction in TαMCA and TβMCA and the increase in secondary BAs (such as LCA and CDCA), through BE administration may have had a profound influence on mouse primary cells, which was in agreement with the results observed *in vivo* and may be responsible for the metabolic improvement effect of BE.

## Discussion

We established a possible pathway involving the GM and BAs to describe how the phenolic compounds in blueberries improve diet- and genetically induced adiposity and its related metabolic syndromes. The daily administration of BE strongly ameliorated the obesity-related disturbance of the GM, thereby regulating the abnormal plasma BA pool size and composition, which further activated the membrane and nuclear BA receptors TGR5 and FXR in BAT, iWAT, and the liver. The activation of TGR5 and FXR increased the energy expenditure in BAT, enhanced the browning of iWAT, and improved lipid metabolism in adipose tissues and the liver, therefore reducing adiposity and improving metabolic syndromes.

BE significantly influenced the abundance of various bacterial genera including *Akkermansia, Bifidobacterium*, *Lactobacillus,* and *Desulfovibrio*. *Bifidobacterium,* and *Lactobacillus*, which produce BSH, were positively correlated with the increased concentration of plasma secondary and free BAs, such as CDCA, DCA, and LCA, and the ratio of secondary/primary BAs. A high ratio of secondary to primary BAs is linked to a decreased risk of non-alcoholic steatohepatitis and a healthy liver status (Molinaro et al., 2018). The high-level expression of BSH in the intestine resulted in reduced weight gain and plasma and hepatic TG concentrations in mice (Joyce et al., 2014), which is consistent with our observations. Although there was no direct evidence that *Akkermansia* produced BSH, *Akkermansia* abundance was correlated with the plasma BA pool, like the abundance of *Bifidobacteria* and *Lactobacillus*. The increase in *Akkermansia* was observed in Roux-en-Y gastric bypass (RYGB) surgery (Cox and Blaser, 2013), which was also associated with profound alteration of the BA pool (e.g., the reduction of taurine-conjugated BAs), the activation of FXR, significant metabolic improvement, and weight loss in the first week after surgery (Ryan et al., 2014, Li et al., 2011). Similar to RYGB, BE treatment ameliorated the HFD-induced increase in plasma taurine-conjugated BAs, including TαMCA and TβMCA, which are inhibitors of FXR and usually present in excess in obese individuals (Molinaro et al., 2018, Sayin et al., 2013), and activated the FXR in the liver. Activation of the FXR in the liver and mouse hepatocytes enhanced the expression of SHP, which further suppressed the activity of SREBP-1c (Watanabe et al., 2004) and its downstream genes related to lipid synthesis including ACC1, AP2, FAS, CD36, and GPAT. The supplementation of CA, an FXR agonist, also lowered TG levels through the activation of the FXR (Watanabe et al., 2004). BE treatment improved glucose metabolism in both wild-type and *db/db* mice, which seems to be related to the activation of the FXR in the liver because treatment of *db/db* mice with the FXR agonist GW4064 decreased hyperglycemia by suppressing the expression of PEPCK and G6Pase (Zhang et al., 2006). Taken together, these results suggest that the activation of the FXR, whether through the reduction of antagonist (RYGB and BE treatment) or the administration of agonist (CA), improved the metabolism in mice.

CA is also a moderate agonist of TGR5 (Perino and Schoonjans, 2015), and treating mice with CA prevented diet-induced obesity and IR (Watanabe et al., 2006). Other kinds of BAs, mainly LCA, DCA, and CDCA, are natural agonists of TGR5 and have effects similar to those of CA (Broeders et al., 2015). Our results showed that BE administration increased the abundance of these free BAs and that treating mouse primary adipocytes derived from BAT with these BAs activated TGR5 in a dose-dependent manner. The activation of TGR5 induces the intracellular accumulation of cyclic AMP, followed by the downstream activation of its signaling pathways, such as the activation of D2 (Thomas et al., 2008). D2 converts the prohormone thyroxine (T_4_) to the active hormone triiodothyronine (T_3_) and increases metabolic rates (Watanabe et al., 2006). Consistent with this, the activation of TGR5 enhanced the expression of a series of downstream genes related to energy expenditure of TGR5 including D2, UCP1, PRDM16, and PGC-1α both *in vivo* and *in vitro*. Antibiotic treatment dramatically decreased the richness of plasma secondary BAs compared with that in the antibiotic-free mice, and the BAT function of antibiotic-treated mice was also lower. However, this decreased BAT activity did not significantly influence BW, which may be because the extinction of the gut bacteria (that were capable of extracting energy from otherwise digested compounds in food) (Kundu et al., 2017) by antibiotics reduced the energy intake of mice from food, as indicated by the increased energy in the feces of antibiotic-treated mice. More importantly, BE administration had no significant effect on the BA pool and the activity of BAT in antibiotic-treated mice, suggesting that gut bacteria played a key role in BE functioning through BAT.

We also found that BE administration significantly reduced the abundance of *Desulfovibrio,* an important H_2_S-producing bacteria in the intestine (Guo et al., 2018). Excess H_2_S can reduce the disulfide bonds in the mucous network and increase the permeability of the intestine, thus contributing to the transposition of bacteria and their metabolites, such as LPS (Ijssennagger et al., 2016). This transposition is able to trigger an inflammatory response and induce IR (Cox et al., 2015). Therefore the reduction in *Desulfovibrio* may be partially conducive to the improvement of inflammation and glucose metabolism in BE-treated mice.

In summary, BE administration improved metabolism by reducing the FXR antagonist content and increasing the abundance of TGR5 agonists, which was fulfilled through regulation of the GM.

### Limitations of the Study

The BE obtained from fresh blueberry fruit was a mixture that consisted of various kinds of phenolic compounds. Therefore, whether there is one or several key ingredients in BE and how BE influences the composition of the GM remains unclear. Moreover, further investigations on GM and BAs are needed to explore the underlying mechanisms of BE-GM-BA-metabolism axis. For example, a full analysis of the BA pools in the plasma, liver, gall bladder, and intestine and the determination of the expressions of BA-related genes would be very helpful to completely understand the influences on BA metabolism by BE treatment. Similarly, sufficient analysis of GM employing metagenomic and metatranscriptomic approaches is also meaningful to have a comprehensive picture of GM alteration. Nevertheless, these results provide a new perspective on how dietary polyphenols function and show the importance of the interaction between the GM and dietary components.

## Methods

All methods can be found in the accompanying Transparent Methods supplemental file.

## Supplemental Information

Document S1. Transparent Methods, Figures S1–S7, and Tables S1 and S2
