# Supplementary material for: Blueberry Extract Improves Obesity through Regulation of the Gut Microbiota and Bile Acids via Pathways Involving FXR and TGR5
Source: iScience. 2019 Aug 16;19:676–90. doi: 10.1016/j.isci.2019.08.020 (PMC6728616; doi:10.1016/j.isci.2019.08.020)
Supplement: Document S1. Transparent Methods, Figures S1–S7, and Tables S1 and S2 [file mmc1.pdf]

ISCI, Volume *19*

Supplemental Information

Blueberry Extract Improves Obesity through Regulation of the Gut Microbiota and Bile Acids via Pathways Involving FXR and TGR5

Jielong Guo, Xue Han, Hongyu Tan, Weidong Huang, Yilin You, and Jicheng Zhan

Table S1 Composition of BE and fecal anthocyanins, related to Figure 1 to Figure 6.

		Extract content (mg/g BE)
	Sugar	15.2 ± 1.23
	Protein	11.0 ± 1.09
	Lipid	2.82 ± 0.23
	Water	76.9 ± 6.10
	Total polyphenols	796.15 ± 36.41
	Unkown	97.60 ± 12.10
Anthocyanins	Total	219.24 ± 23.26
	Cyanidin-3-hexosides	13.31 ± 0.81
	Delphinidin-3-hexosides	56.77 ± 5.63
	Petunidin-3-hexosides	36.73 ± 2.59
	Malvidin-3-hexosides	60.9 ± 5.52
Proanthocyanidins		161.8 ± 23.85
Phenolic acids	Total	77.91 ± 9.82
	caffeic acid	2.37 ± 0.34
	p-Coumaric acid	13.52 ± 1.28
	Ferulic Acid	10.08 ± 1.17
	4-Hydroxybenzoic acid	21.99 ± 2.38
	Gallic acid	6.83 ± 0.91
	Protocatechuic acid	6.75 ± 0.84
	Vanillic acid	7.87 ± 0.92
Flavanols/flavonols	Total	101.88 ± 12.46
	Quercetin-3-hexosides	37.04 ± 5.93
	Myricetin-hexosides	48.91 ± 5.82
	Kaempferol-hexosides	6.79 ± 0.81

Fecal anthocyanins contents (mg/g)

mg/g feces	Cyanidin-3-hexosides	Delphinidin-3-hexosides	Petunidin-3-hexosides	Malvidin-3-hexosides	Total
BE1	ND	0.27 ± 0.12^a	0.19 ± 0.11^a	0.29 ± 0.17^a	0.75 ± 0.37^a
BE2	0.19 ± 0.13^a	0.31 ± 0.15^a	0.26 ± 0.17^a	0.24 ± 0.13^a	1.00 ± 0.42^a
BE3	0.14 ± 0.08^a	0.34 ± 0.13^a	ND	0.33 ± 0.19^a	0.82 ± 0.31^a
ABE	1.09 ± 0.58^b	2.49 ± 1.21^b	1.51 ± 1.36^b	2.33 ± 1.91^b	7.42 ± 2.09^b

Data are presented as the mean ± SD.

Hexosides represent glucoside, galactoside and arabinoside.

Data labeled with different letters have significant difference, $P < 0.05$.

Table S2 Diet composition, related to Figure 1 to Figure 6.

	**D12450B**		**D12492**	
	gm%	*kcal%*	gm%	*kcal%*
Protein	19.2	*20*	26.2	*20*
Carbohydrate	67.3	*70*	26.3	*20.1*
Fat	4.3	*10*	34.9	*59.9*
Total		*100*		*100*
kcal/gm	3.85		5.24	
**Ingredient**	**gm**	***kcal***	**gm**	***kcal***
Casein	200	*800*	200	*800*
L-Cystine	3	*12*	3	*12*
Corn Starch	315	*1260*	0	*0*
Maltodextrin 10	35	*140*	125	*500*
Sucrose	350	*1400*	68.8	*275.2*
Cellulose, BW200	50	*0*	50	*0*
Soybean Oil	25	*225*	25	*225*
Lard	20	*180*	245	*2205*
Mineral Mix S10026	10	*0*	10	*0*
DiCalcium Phosphate	13	*0*	13	*0*
Calcium Carbonate	5.5	*0*	5.5	*0*
Potassium Citrate, 1 H_2O	16.5	*0*	16.5	*0*
Vitamin Mix V10001	10	*40*	10	*40*
Choline Bitartrate	2	*0*	2	*0*
FD&C Yellow Dye #5	0.05	*0*	0	*0*
FD&C Red Dye #40	0	*0*	0	*0*
FD&C Blue Dye #1	0	*0*	0.05	*0*
**Total**	**1055.05**	***4057***	**773.85**	***4057***

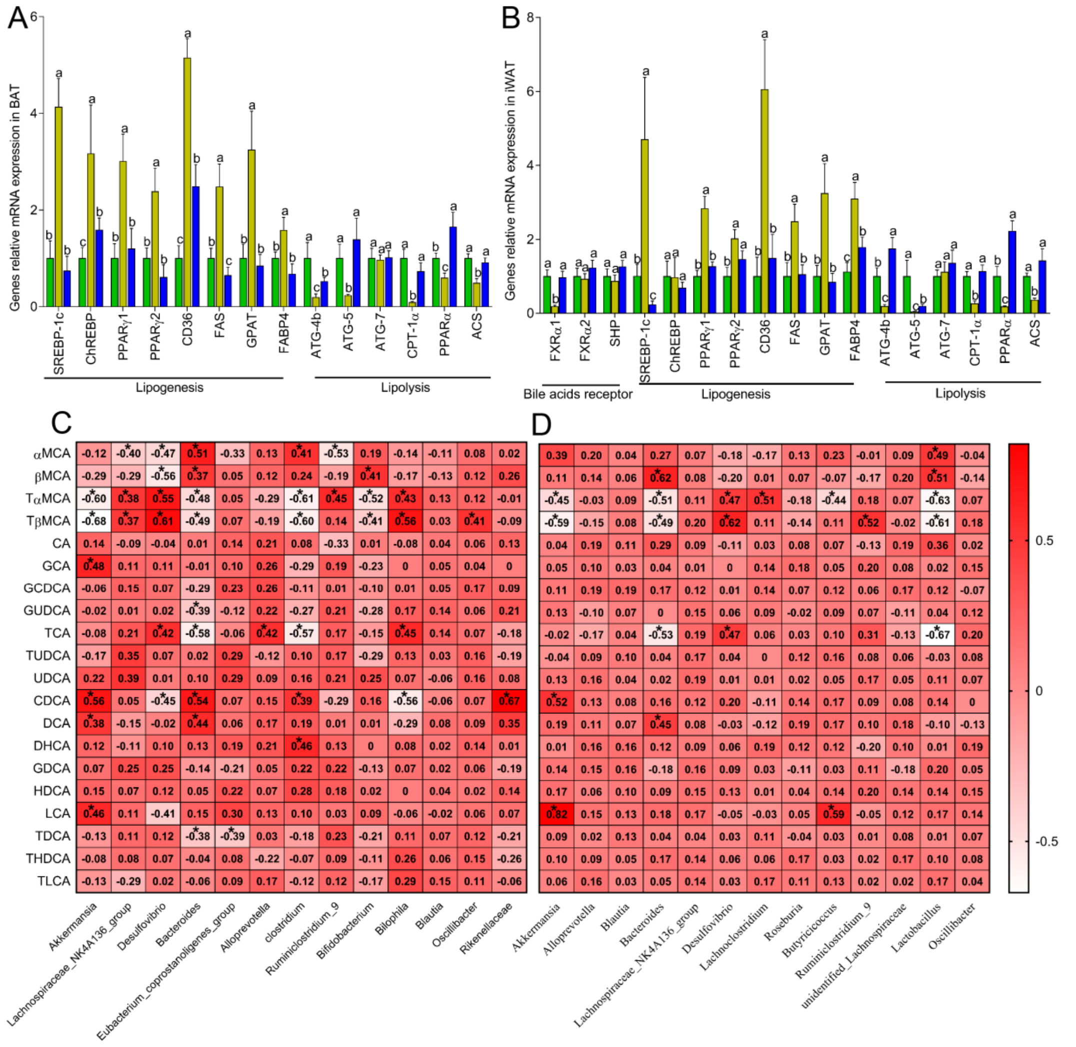

Figure S1 BE administration improved lipid metabolism in BAT and iWAT and the correlation heatmap between the most abundant bacterial genera and the plasma bile acids, related to Figure 3 and 5. The relative mRNA expression of genes in BAT (**A**) and iWAT (**B**) showed that BE administration suppressed the lipogenesis while enhanced lipolysis in BAT and iWAT. Correlation heatmaps between the most abundant bacteria taxa and the plasma bile acids of mice from study1 (**C**) and study2 (**D**) showed that there were strong correlations exiting. Significance and r values were according to Spearman (*$P < 0.05$* if 0.362 < r < 0.467; *$P < 0.01$* if 0.467 < r < 0.580; *$P < 0.001$* if r > 0.580 for (**C**) and *$P < 0.05$* if 0.435 < r < 0.556; *$P < 0.01$* if 0.556 < r < 0.681; *$P < 0.001$* if r > 0.681 for (**D**)).

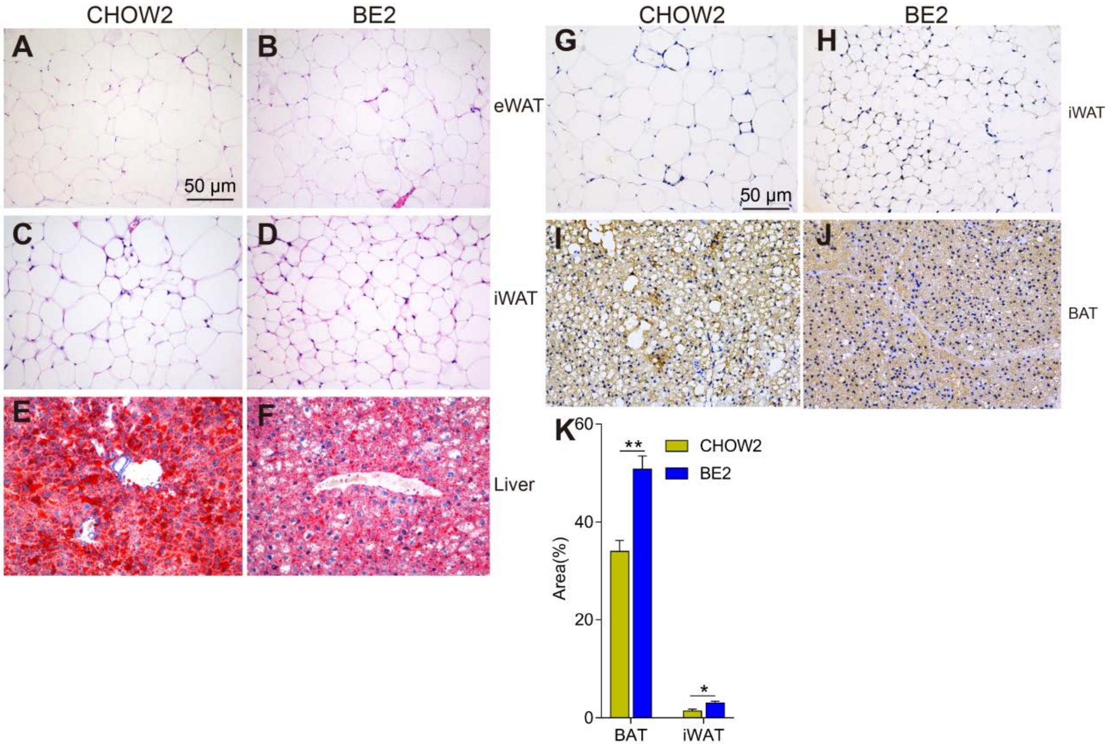

Figure S2 BE treatment decreased the fat deposition in WAT and liver, related to Figure 5. (**A**–**D**) Representative H&E staining of eWAT and iWAT of mice from CHOW2 and BE2 groups showed that BE treatment reduced fat deposition in WAT as well as the size of adipocytes, n = 10. (**E** and **F**) Representative oil red O staining of liver of mice from CHOW2 and BE2 groups, red areas indicate the lipid, n = 10. BE treatment enhanced the browning of iWAT (**G** and **H**) and the expression of UCP1 in BAT (**I** and **J**). (**K**) Area percent of the stained parts.

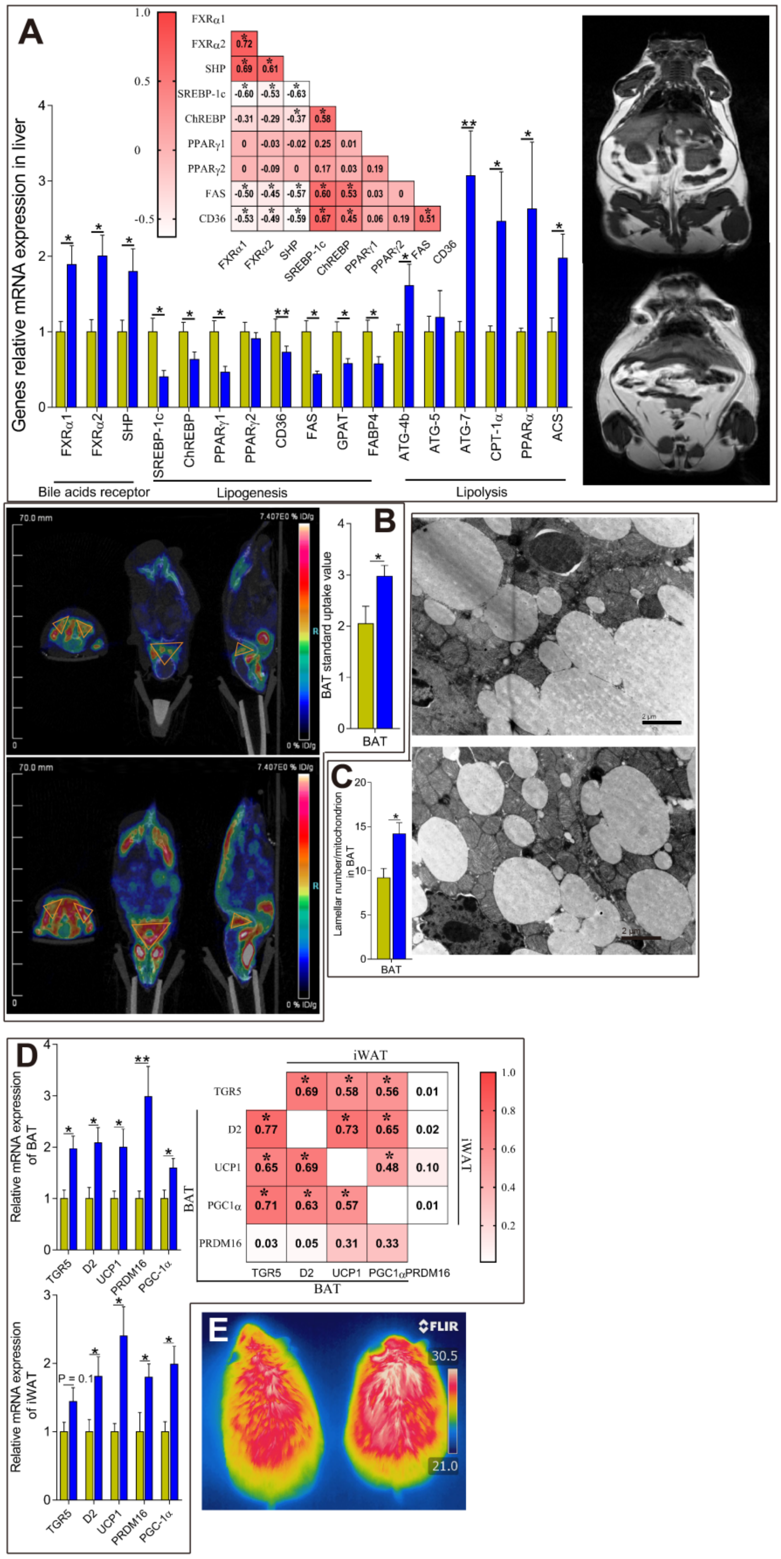

Figure S3 BE administration improves genetically-induced obesity and enhanced energy expenditure of *db/db* mice, related to Figure 5. (**A**) The relative mRNA expression of genes in liver (left bottom) and representative MRI images (right) (upper, CHOW2; lower, BE2); white areas indicate lipids, n = 8. Upper left, correlation matrix between the mRNA expression levels of key genes controlling lipogenic and BA receptors;

r values and significance were according to Spearman’s rank correlation test (**P* < *0.05* if 0.435 < r < 0.556; **P* < *0.01* if 0.556 < r < 0.681; **P* < *0.001* if r > 0.681). (**B**) Representative PET/CT scan (upper left, CHOW2; lower left, BE2) of mice after mild cold stimulation. Yellow triangles indicate the anatomical site of the interscapular BAT, n = 5. (**C**) Representative transmission electronic microscopy images (upper right, CHOW2; lower right, BE2) of BAT. Scale bar, 2 μm, original magnification 12,000×, n = 8. (**D**) The mRNA expression of genes related to the activation of BAT (upper left) and the browning of iWAT (bottom left). Right, correlation matrix between the mRNA expression of genes in BAT and iWAT; r value and significance were according to Spearman’s rank correlation test (**P* < *0.05* if 0.435 < r < 0.556; **P* < *0.01* if 0.556 < r < 0.681; **P* < *0.001* if r > 0.681). (**E**) Representative infrared thermal images of mice in BE2 (right) and CHOW2 groups (left). For all figures, **P* < *0.05*, ***P* < *0.01* and ****P* < *0.001*.

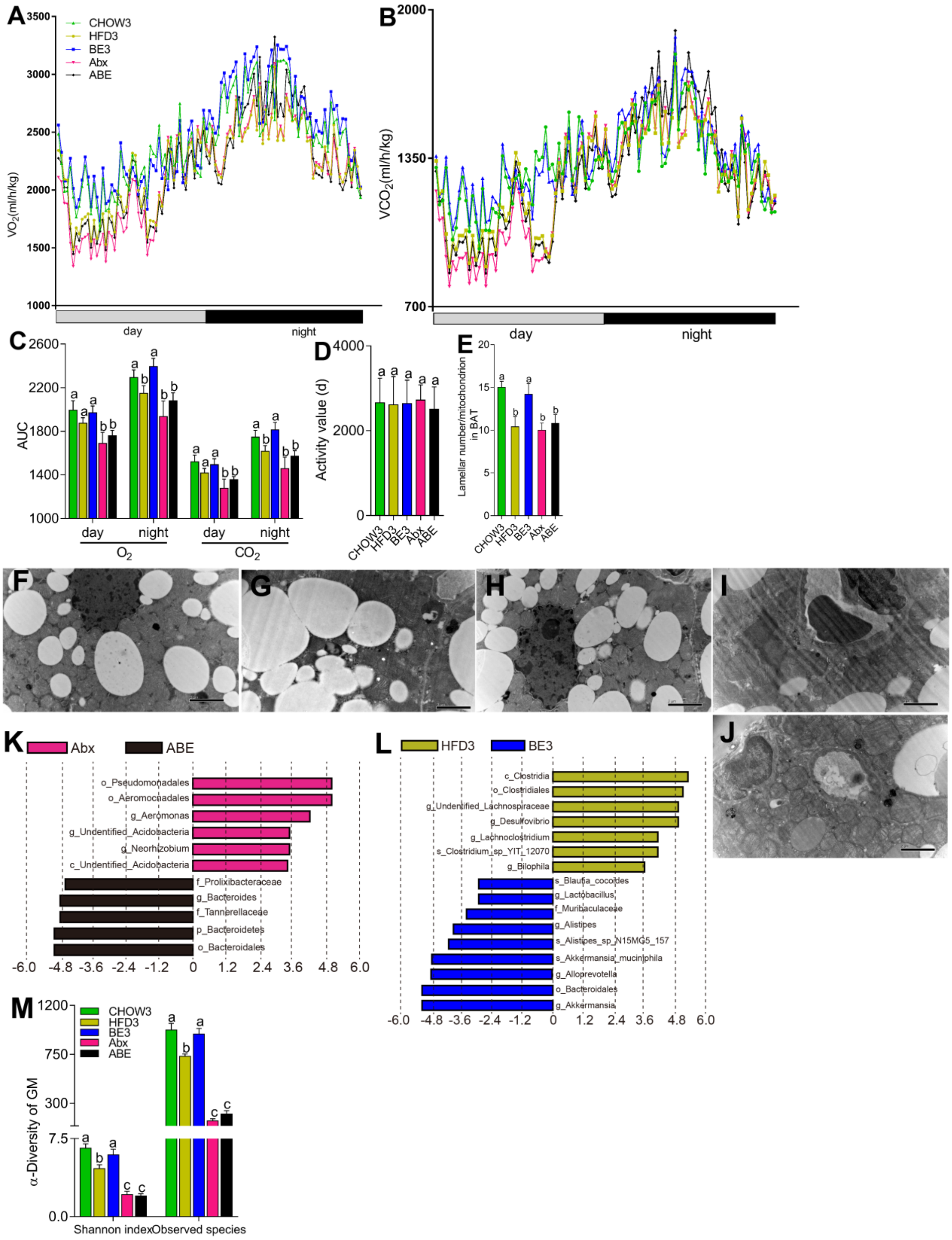

Figure S4 **Antibiotics treatment blunted the increase of energy expenditure and the regulation of GM in BE-treated mice, related to Figure 6**. (**A**, **B** and **C**) The daily O_2 consumption (**A**), CO_2 production (**B**) of mice and the relative AUC (**C**). Columns indicated with different letters have significant difference, $P < 0.05$. (**D**) The daily activity of mice showed that the discrepancy on energy expenditure was not related to the physical activity. (**F**-**J**) Representative transmission electron microscope images of mitochondria in BAT of mice belong to CHOW3, HFD3, BE3, Abx and ABE groups, respectively. (**E**) Quantification of the lamellar number in BAT, n = 9-12. (**K** and **I**) Biomarkers identified by

LEfSe between Abx and ABE (**K**), HFD3 and BE3 (**l**) groups. (**M**) The Shannon index and observed species of GM. For all pictures, columns indicated with different letters have significant difference, $P < 0.05$.

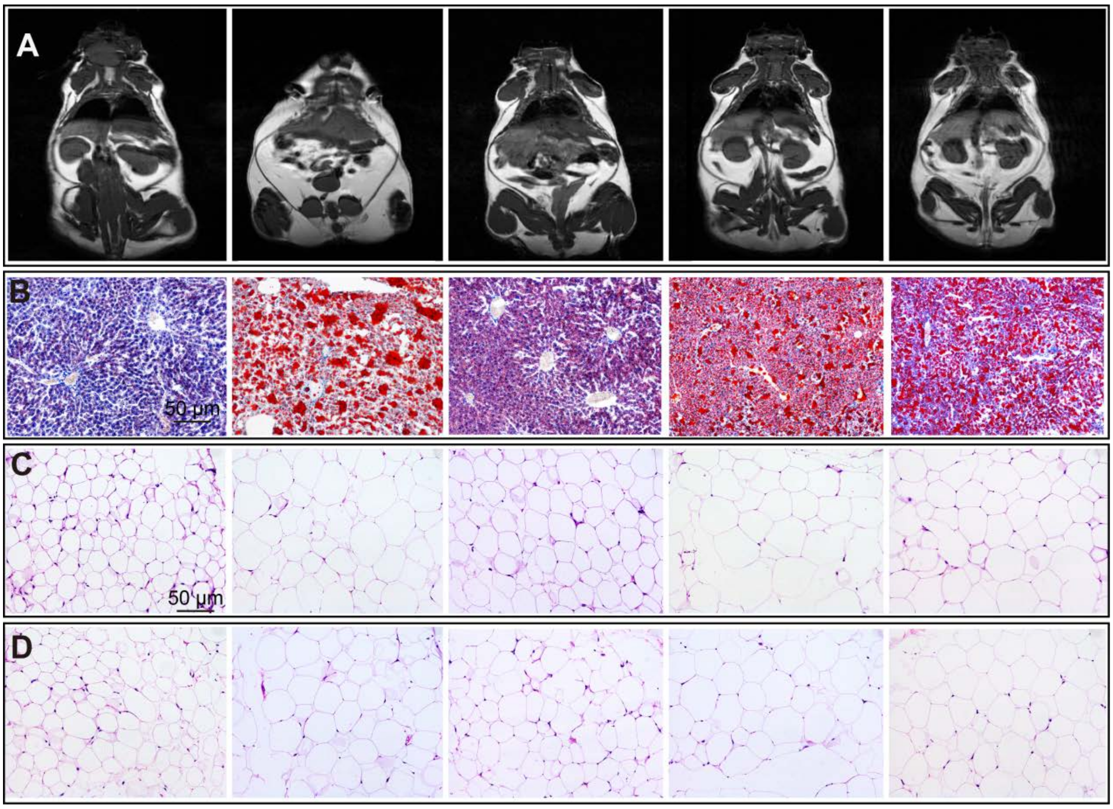

Figure S5 Antibiotics blunted the improvement of adiposity and fat deposition in liver and WAT upon BE administration, related to Figure 6. (**A**) Representative MRI images, white part represent the lipid. (**B**) Representative oil red O staining images of liver, red part represent the lipid. (C) Representative H&E staining images of eWAT. (**D**) Representative H&E staining images of iWAT. For all treatments, images represent the mice of CHOW3, HFD3, BE3, Abx and ABE groups, respectively, from left to right, n = 9 – 12.

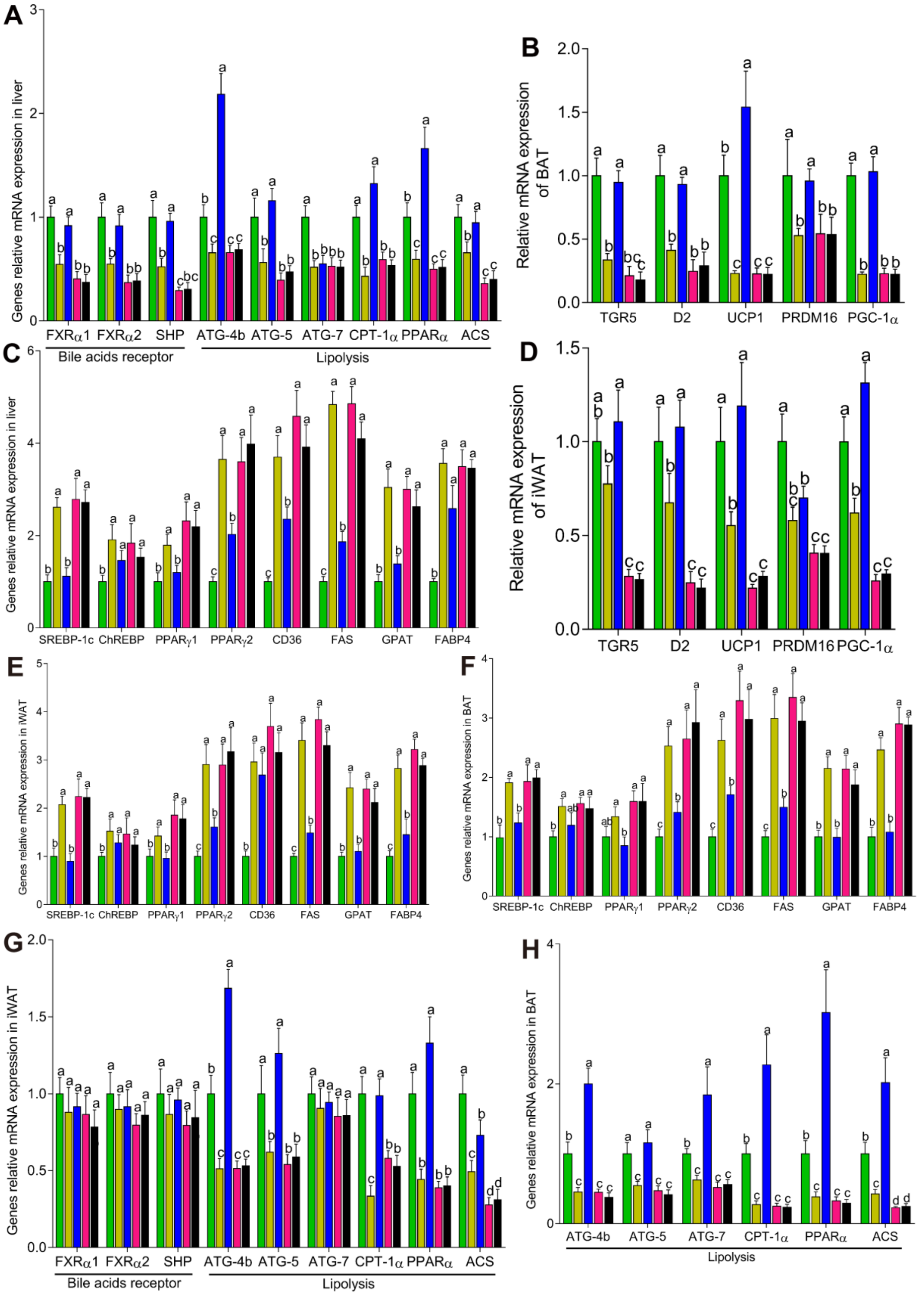

Figure S6 Antibiotics treatment totally blunted the metabolic regulation effect of BE in liver, iWAT and BAT, related to Figure 6. (**A**, **C and E-H**) BE administration enhanced the mRNA expression of FXR and SHP, elevated the mRNA expression of genes related with lipolysis (**A**) and suppressed the mRNA expression of SREBP-1c and its' downstream genes related to lipogenesis in liver (**A**), which were totally blunted by antibiotics treatment and similar results were observed in iWAT (**E** and **G**) and BAT (**F** and **H**). (**B** and **D**) The enhancement of the activation of BAT and the browning of iWAT in BE treated mice were

blunted by antibiotics treatment. For all pictures, columns indicated with different letters have significant difference, $P < 0.05$.

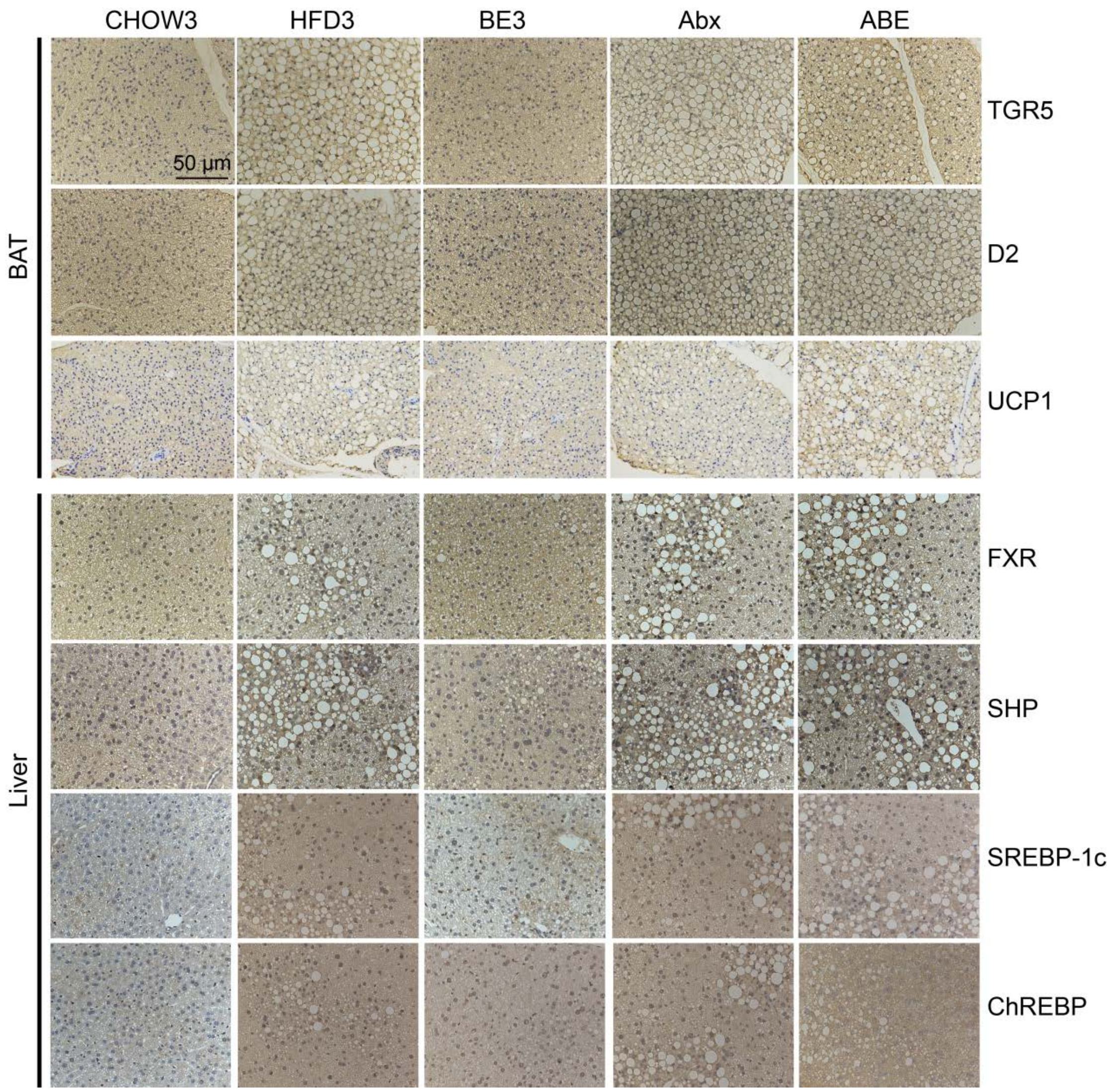

Figure S7 Antibiotics treatment blunted the regulation of protein expression related to NST in BAT and lipogenesis in liver, related to Figure 6. Representative immunohistochemistry images for proteins related to NST in BAT and lipogenesis in liver showed that BE administration enhanced NST and suppressed lipogenesis, which were blunted by antibiotics treatment, n=9-12

Methods

KEY RESOURCES TABLE

REAGENT or RESOURCE	SOURCE	IDENTIFIER
Antibodies		
Anti-GAPDH	Abcam	AB_11129118
Anti-FXR1	Abcam	AB_11154960
Anti-SHP1	Abcam	AB_777912
Anti-SREBP1	Abcam	AB_777912
Anti-CHREBP	Abcam	AB_10562135
Anti-TGR5	Abcam	AB_2112165
Anti-Dio2 (D2)	Sigma-Aldrich	AB_10600804
Anti-UCP1 [EPR20381]	Abcam	AB_2722676
Anti-PGC 1 alpha	Abcam	AB_881987
Anti-mouse MUC2	Abcam	AB_10713220
Chemicals		
ampicillin	Sigma-Aldrich	Cat#171254-25GM
metronidazole	Sigma-Aldrich	Cat#M3761-25G
gentamicin	Sigma-Aldrich	Cat# E003632-10G
neomycin	Sigma-Aldrich	Cat# N6386-25G
vancomycin	Sigma-Aldrich	Cat# V2002-5G
CA	Sigma-Aldrich	Cat# C1129-25G
CDCA	Sigma-Aldrich	Cat# C9377-5G
LCA	Sigma-Aldrich	Cat# L6250-10G
DCA	Sigma-Aldrich	Cat# D6750-10G
TβMCA	Steraloids Inc	Cat# C1899-000
Critical Commercial Assays		
QIAamp DNA Stool Mini Kit	QIAGEN	Cat# 51504
Bile Acid Assay Kit	Sigma-Aldrich	Cat# MAK309
Mouse Leptin ELISA Kit	Sigma-Aldrich	Cat# RAB0334-1KT
TNF alpha Mouse ELISA Kit	Invitrogen	Cat# BMS607-3
Mouse IL-6 ELISA Kit	Sigma-Aldrich	Cat# RAB0308-1KT
Mouse LPS ELISA Kit	CUSABIO	Cat# CSB-E13066m
Software and Algorithms		
Prism 7.00	GraphPad	N/A
SPSS Statistics 20	IBM	N/A

Oligonucleotides

Gene	FORWARD PRIMER (5'-3')	REVERSE PRIMER (5'-3')	REF	SOURCE
Fungi ITS1	CTTGGTCATTTAGAGGAAGTAA	GCTGCGTTCTTCATCGATGC	1	Thermo Fisher
Universal	AGAGTTTGATCCTGGCTCAG	CTGCTGCCTCCCGTAGGAGT	2	Thermo Fisher
Bacteroidetes	GGARCATGTGGTTTAATTCGATGAT	AGCTGACGACAACCATGCAG	2	Thermo Fisher
Firmicutes	GGAGYATGTGGTTTAATTCGAAGCA	AGCTGACGACAACCATGCAC	2	Thermo Fisher
Akkermansia	CAGCACGTGAAGGTGGGGAC	CCTTGCGGTTGGCTTCAGAT	3	Thermo Fisher
Bilophila	CGGTATCGAAATCGTGAAGG	CAGAGGGTCAGGGTGTTGTT	4	Thermo Fisher
Desulfovibrio	CCGTAGATATCTGGAGGAACATCAG	ACATCTAGCATCCATCGTTTACAGC	5	Thermo Fisher
Bifidobacterium	GGGTGGTAATGCCGGATG	CCACCGTTACACCGGGAA	6	Thermo Fisher
Lactobacillus	AGCAGTAGGGAATCTTCCA	ATTYCACCGCTACACATG	6	Thermo Fisher
TJP1	TTTTTGACAGGGGGAGTGG	TGCTGCAGAGGTCAAAGTTCAAG	7	Thermo Fisher
Occludin	ATGTCCGGCCGATGCTCTC	TTTGGCTGCTCTTGGGTCTGTAT	8	Thermo Fisher
MUC2	TGTGGCCTGTGTGGGAACTTT	CATAGAGGGCCTGTCCTCAGG	7	Thermo Fisher
TLR4	GCAGAAAATGCCAGGATGATG	AACTACCTCTATGCAGGGATTCAAG	7	Thermo Fisher
TNFα	CGAGTGACAAGCCTGTAGCC	CATGCCGTTGGCCAGGA	8	Thermo Fisher
IL-6	TCCATCCAGTTGCCTTCTTG	TTCCACGATTTCCCAGAGAAC	8	Thermo Fisher
F4/80	CCCAGCTTATGCCACCTGCA	TCCAGGCCCTGGAACATTGG	9	Thermo Fisher
CEBPβ	GTTCTTGACGTTCTTCGGCCG	TGGACAAGCACAGCGACGAGT	10	Thermo Fisher
CEBPσ	AGTTTCTTGGGACATAGGAGCGCA	GTACCTTAGCTGCATCAACAGGAG	11	Thermo Fisher
CYP7A1	AGGACTTCACTCTACACC	GCAGTCGTTACATCATCC	12	Thermo Fisher
IBAT	GCCCCAAAAAGCAAAGATCA	GCTATGAGCACAATGAGGATGG	13	Thermo Fisher
FXRα1	CATCAGAGTACTTGAAGAAGATGTTGTCAGAGGACGG	CTCCTTCCGACATGGAGAAGTCATCGC	14	Thermo Fisher
FXRα2	GAAGGGGAGATGGTTTTGTTGTAAAGGCTCAAAG	TCTTGAAGACTGCATCTGTACCAGGGCCAT	14	Thermo Fisher
SHP	CCCTCCACAAACAATCCAGCCTTT	CATTAAGAGCCTGCCTGCGTTCAA	14	Thermo Fisher
SREBP-1c	CCAGCGGCTGCCTTCACACA	CCAGCCGAAAAGCGAGGCCA	15	Thermo Fisher
ChREBP	CGGGACATGTTTGATGACTATGTC	CATCCCATTGAAGGATTCAAATAAA	16	Thermo Fisher
PPARg1	GAGTGTGACGACAAGATTTG	GGTGGGCCAGAATGGCATCT	17	Thermo Fisher
PPARg2	TCTGGGAGATTCTCCTGTTGA	GGTGGGCCAGAATGGCATCT	17	Thermo Fisher
CD36	GGAACTGTGGGCTCATTGC	CATGAGAATGCCTCCAAACAC	2	Thermo Fisher
FAS	TGATGTGGAACACAGCAAG	GGCTGTGGTGACTCTTAGTGATAA	2	Thermo Fisher
GPAT	TCTGCTGCCATCTTTGTCCAC	TTGGTCTCTTTGAAAACCCCG	2	Thermo Fisher
FABP4	TTAAAAACACCGAGATTTCCTTCAA	GGGCCCCGCCATCTAG	16	Thermo Fisher
ATG-4b	TGGGTGTTATTGGAGGGAAG	CAGAAAAACCCCACAGCAAT	15	Thermo Fisher
ATG-5	TAGAATATATCAGACCACGACG	CTCCTCTTCTCTCCATCTTC	15	Thermo Fisher
ATG-7	TCCGTTGAAGTCCTCTGCTT	CCACTGAGGTTCACCATCCT	15	Thermo Fisher
CPT-1α	AGCACACCAGGCAGTAGCTT	AGGATGCCATTCTTGATTCG	18	Thermo Fisher
PPARα	GCGTACGGCAATGGCTTTAT	GAACGGCTTCCTCAGGTTCTT	15	Thermo Fisher
ACS	TATCATGCTTCACCTATGGC	CAAATAAGAGGAGCTCCAAC	19	Thermo Fisher
TGR5	GAGCGTCGCCCACCACTAGG	CGCTGATCACCCAGCCCCATG	20	Thermo Fisher
D2	CTTCCTCCTAGATGCCTACAAAC	GGCATAATTGTTACCTGATTCAGG	21	Thermo Fisher
UCP1	AGGCTTCCAGTACCATTAGGT	CTGAGTGAGGCAAAGCTGATTT	22	Thermo Fisher
PGC-1α	CCCCTTTGCCCTGACCTGCCTGAG	GAAGGACAGCTCTGATCACTGGCATTGG	23	Thermo Fisher
PRDM16	CAGCACGGTGAAGCCATTC	GCGTGCATCCGCTTGTG	22	Thermo Fisher

β-actin	GGATGCAGAAGGAGATCACTG	CGATCCACACGGAGTACTTG	24	Thermo Fisher

The bile acids standards including α-murocholic acid (αMCA), β-murocholic acid (βMCA), tauro-α-murocholic acid (TαMCA), tauro-β-murocholic acid (TβMCA), tauro-ursodesoxycholic acid (TUDCA), taurocholic acid (TCA), tauro-ursodeoxycholic acid (TDCA), glycocholic acid (GCA), glycodeoxycholic acid (GDCA), Cholic acid (CA), ursodeoxycholic acid (UDCA), hyodeoxycholic acid (HDCA), chenodeoxycholic acid (CDCA), deoxycholic acid (DCA), glycochenodeoxycholic acid (GCDCA), glycoursodeoxycholic acid (GUDCA), taurohyodeoxycholic acid (THDCA), taurolithocholic acid (TLCA), dehydrocholic acid (DHCA), lithocholic acid (LCA), [2,2,4,4-D4]-DCA (DCA-d4), [2,2,4,4-D4]-CA (CA-d4), [2,2,4,4-D4]-CDCA (CDCA-d4), [2,2,4,4-D4]-LCA (LCA-d4), [2,2,4,4-D4]-GCDCA (GCDCA-d4), [2,2,4,4-D4]-GCA (GCA-d4) were all purchased from Steraloids Inc (Newport, Rhode Island, USA).

CONTACT FOR REAGENT AND RESOURCE SHARING

Further information and requests for resources and reagents should be directed to and will be fulfilled by the Lead Contact, Zhan Jicheng (zhanjicheng@cau.edu.cn).

EXPERIMENTAL MODEL AND SUBJECT DETAILS

Animals

We performed three independent mouse studies to study the effect of BE on adiposity and its related metabolic syndromes based on high fat diet (HFD)- (studies 1 and 3) and genetically- (study 2)-induced obesity models. In study 1, male C57BL/6J mice purchased at 21 days of age (Vital River Laboratory Animal Technology. Co., Ltd., China) were randomly assigned to four groups (n = 9-12) after adapting for one week as follows: (1) a CHOW1 group fed a chow diet (3.85 kcal/g, 10% energy from fat) (D12450B, Research Diets, USA), (2) a HFD1 group fed a HFD (4.73 kcal/g, 60% energy from fat) (D12492, Research Diets), (3) a CBE group (5 gL^{-1} BE in drinking water) fed a chow diet, and (4) a BE1 group (0.5% (m/v) BE in drinking water) fed a HFD. In study 2, male C57BL/KsJ *db/db* mice purchased at 21 days of age (Model Animal Research Center of Nanjing University, Nanjing, China) were randomly assigned to two groups (n = 10) as follows: (1) a CHOW2 group fed a chow diet and (2) a BE2 group fed a chow diet with 5 gL^{-1} BE in drinking water. In study 3, male C57BL/6J mice purchased at 49 days of age (Vital River Laboratory Animal Technology. Co., Ltd.) were randomly assigned to five groups (n = 10-12) as follows: (1) a CHOW3 group fed a chow diet, (2) a HFD3 group fed a HFD, (3) a BE3 group (5 gL^{-1} BE in drinking water) fed a HFD, (4) an Abx group fed a HFD and (5) an ABE group (5 gL^{-1} BE in drinking water) fed a HFD. In study 3, all the mice were gavaged daily with 200 μL PBS containing (for the Abx and ABE groups) 0.5 mg ml^{-1} ampicillin, 0.5 mg ml^{-1} gentamicin, 0.5 mg ml^{-1} metronidazole, 0.5 mg ml^{-1} neomycin and 0.25 mg ml^{-1} vancomycin[25]

(Sigma-Aldrich, USA) or no antibiotics (for the CHOW3, HFD3 and BE3 groups).

Mice in all three studies were housed 3/cage in standard specific pathogen-free (SPF) conditions (12/12 h light/dark cycle, humidity of 50 ± 15%, temperature of 22 ± 2 ℃) and were allowed to adapt for one week before the experiments were started. The food used in these studies was sterilized using radiation (25.0 kGy). Food and water were provided *ad libitum,* and food intakes were recorded every week. Body weights were recorded weekly, the drinking water was changed every three days and fecal samples were collected at intervals. Mice eighteen weeks of age were fasted for 12 h in all mouse studies, plasma was dried in tubes following eyeball extirpation, and the mice were killed through breaking their necks immediately after taking the blood. After standing for 1 h, the blood was centrifuged at 3000 rpm for 10 min at 4℃ to obtain the blood plasma. The liver, inguinal white adipose tissue (iWAT), epididymal white adipose tissue (eWAT), brown adipose tissue (BAT), skeletal muscle, ileum and colon were obtained, weighed and placed into a 4% aqueous paraformaldehyde solution or liquid nitrogen immediately and then stored at -80℃ or room temperature for further analysis. The contents of the ileum, caecum, colon and rectum were collected and stored at -80℃ for GM analysis.

The guidelines of the National Institutes of Health regarding the care and use of laboratory animals were followed. This study was approved by the Animal Experiment Committee of the College of Food Science and Nutritional Engineering at China Agricultural University.

Cell Separation and Culture

The primary adipocytes were separated from the interscapular BAT of 5- to 6-week-old male mice, and the primary hepatocytes were separated from 8-week-old male mice using collagenase digestion and perfusion methods, respectively. Adipocyte differentiation was induced by treating confluent cells in DMEM (D-glucose, 25 mM) containing 10% fetal bovine serum (FBS), 0.5 mM isobutylmethylxanthine, 125 nM indomethacin, 1 mM dexamethasone, 850 nM insulin, 1 nM T3, 1 lM rosiglitazone, 100 U/ml penicillin, and 100 mg/ml streptomycin. After 2 days of induction, the cells were maintained in differentiation media (10% FBS, 850 nM insulin, 1 nM T3 and 1 lM rosiglitazone) in cell culture plates for the following experiment.[26] Hepatocytes were maintained in DMEM/F-12 (Gibco) with 10% FBS (Gibco), 1% ITS Liquid Media Supplement (Gibco), 40 ng/ml dexamethasone (Sigma), and 1% pen/strep (Gibco) in cell culture plates. All plates were incubated at 37℃ in 5% CO_2.

Adipocytes were treated with different concentrations (3 μM, 30 μM or 300 μM) of CDCA or LCA for 24 h, and then the cells were collected for mRNA expression and western blotting analysis. Hepatocytes were treated with or without CDCA (50 μM) and different concentrations (0, 100 μM or 300 μM) of TαMCA and TβMCA for 12 h for mRNA expression analysis and 24 h for western blotting analysis.

METHOD AND DETAILS

Extract and Analysis of the Phenolic Compounds in BE and Feces

The blueberries used in this study were highbush blueberries (*Vaccinium corymbosum*) grown in

the Daxing District, Beijing, China and harvested in 2016. The extraction and analysis of phenolic compounds from blueberries were performed as previously described with some modification.[27] Briefly, blueberry fruit or fresh feces was macerated and stirred with 95% ethanol (1:5, w (g)/v (ml)). The extract was then purified through an Amberlite XAD-7 column. The phenolic compounds were eluted with moderate absolute ethanol with 1% (v/v) formic acid. The eluent was concentrated under reduced pressure at 35°C and freeze-dried.

The total polyphenol content in the BE was determined with a modified Folin-Ciocalteu method, and the total anthocyanin content of the BE was directly determined using a pH differential method as previously described.[27] The results are presented as mg gallic acid or cyanidin-3-O-glucoside equivalent/100 mg of BE, respectively.

The concentrations of phenolic compounds in the BE and anthocyanins in the feces were determined with a UPLC (Waters, Milford, MA, USA) equipped with a QqQ-MS and diode array detector (DAD) as described with some modifications.[28] Chromatographic separations were performed on an Acquity UPLC HSS T3 (Waters) column (2.1× 100 mm, 1.7 μm). The injection volume was 2 μL, and a 0.4 mL/min flow rate was used. The mobile phase consisted of 2 phases: (A) and (B). Mobile phase (A) was 5% (v/v) formic acid in water, and mobile phase (B) was 5% (v/v) formic acid in acetonitrile. BE and FE were dissolved in mobile phase (A) and filtered through a 0.22 μm membrane filter. The elution conditions were as follows: 0~0.5 min, 99% A; 0.5~16 min, 99~65% A; 16~18 min, 65~0% A; 18~21 min, 0% A; 21~23 min, 0~99% A; 23~23.5 min, 99% A. The column temperature was 40°C, and the detection wavelength was 520 nm (for the determination of anthocyanins).

Determination of the Composition and Concentration of Bile Acids in the Plasma through LC-MS

The plasma composition and concentration of twenty common bile acids were determined through UPLC/electrospray ionization mass spectrometry (UPLC/ESI-MS) as described with some modification.[29] Briefly, one milliliter of precooled ethanol was added to a 50 μL plasma sample. Following vortex and centrifugation, the supernatant was then filtered with a 0.22 μm filter before analysis with LC-MS.

Liquid chromatography (LC) separation was performed using a Waters Acquity UPLC system with a BEH C18 column (1.7 μm, 100 mm, 2.1 mm; Waters) and maintained at 40°C. The sample injection volume was 1 μL. Solvent A was water containing 0.005% (v/v) formic acid. Solvent B was acetonitrile containing 0.005% formic acid, and the flow rate was 0.4 mL/min. The gradient program was as follows: 0 ~ 2 min, 77% ~ 67% A; 2 ~ 6 min, 67% ~ 66% A; 6 ~ 11 min, 66% ~ 30% A. MS analysis was performed using a Quattro Premier XE quadrupole tandem MS (Waters) equipped with an ESI probe in negative-ion mode. A capillary voltage of -3,200 V, a source temperature of 120°C, and a desolvation temperature of 400°C were used. The concentrations of individual BAs were calculated from the peak area in the chromatogram detected with selected ion recording relative to that of the internal standard, nordeoxycholic acid.

Quantitative Real-time PCR (qPCR) Analysis

Total RNA was extracted using TRIzol™ reagent (Invitrogen) according to the manufacturer's instructions. Reverse transcription of the total RNA (2.5 μg) was performed with a high-capacity cDNA reverse transcription kit (Promega Biotech Co., Ltd). qPCR was run in triplicate for each sample and analyzed in a LightCycler 480 real-time PCR system (Roche). Data were normalized to the internal control *β-actin* and analyzed using the △△CT method.[30] The expression of genes related to lipid metabolism (in the WAT, BAT and liver), inflammation (in the WAT and liver), permeability (in the ileum and colon), and thermogenesis (in the BAT), as well as the bacterial load, were determined through qPCR (the related genes and primers used are shown in Table S1).

Quantification of the bacterial load through qPCR was conducted as previously described.[30] Briefly, the total bacterial DNA was isolated from the samples with a QIAamp DNA Stool Mini Kit (Qiagen). The DNA was then subjected to qPCR using a QuantiFast SYBR Green PCR kit (Bio-Rad) with universal 16S rRNA primers (Table 1).[2] The results are expressed as bacterial number per g of sample using a standard curve made using *Bifidobacterium longum*.

Metabolic Rate and Physical Activity

Oxygen consumption and physical activity were determined in 16-week-old mice before a GTT. Oxygen consumption was measured using TSE lab master systems as previously described.[22] All mice were acclimatized for 24 h prior to the measurements, and then VO_2 and VCO_2 were measured over the course of 24 h. The mice were maintained at 25°C under a 12 h light/dark cycle with free access to food and water. Their physical activity was measured by the optical beam technique (Opto-M3; Columbus Instruments, Columbus, OH, USA) over 24 h and calculated as 24 h average activity.

Positron Emission Tomography-Computed Tomography and Transmission Electron Microscopy

At the end of the experiment, the mice fasted overnight. After exposure to a cold environment (4°C) for 30 min, the mice were lightly anesthetized with isoflurane and injected with 18F-FDG (500 mCi) via the tail vein. Sixty minutes after radiotracer injection, the mice were subjected to PET/CT imaging with the Siemens Inveon Dedicated PET (dPET) system and an Inveon Multimodality System (CT/SPECT) (Siemens Preclinical Solutions, Knoxville, TN, USA) at the Institute of Laboratory Animal Sciences, Chinese Academy of Medical Sciences. Inveon Acquisition Workplace software was used for the scanning process. The positron emission tomography-computed tomography (PET-CT) instrument parameters were as previously described, and data analysis was performed as previously described.[31]

BAT sections were fixed with 2% (v/v) glutaraldehyde in 0.1 M phosphate buffer (pH 7.4) for 12 h at 4°C. The sections were then postfixed with 1% osmium tetroxide, dehydrated in ascending concentrations of ethanol, and embedded in fresh epoxy resin 618. Ultrathin sections (60–80 nm) were cut and stained with lead citrate before being examined on a Hitachi H-7500 transmission electron microscope.

MRI

MRI experiments were performed on 15-week-old mice. MRI measurements were performed on a 7.0 T Varian MRI instrument (Varian Medical Systems, Palo Alto, CA, USA) using a 40 mm volume and receiver coil at the Institute of Laboratory Animal Sciences, Chinese Academy of Medical Sciences. Prior to the experiments, the mice were initially anesthetized with 2% isoflurane in a dedicated chamber. During the course of MRI, anesthesia levels were reduced to 1.5–1% in a combination of medical air and medical oxygen. The mice were positioned in the prone position, and respiratory-gated image acquisition was performed. MRI images of the mice were analyzed by Argus software.

Glucose and Insulin Tolerance Tests (GTT and ITT)

A GTT was performed on 16-week-old mice after a 16 h fast. Glucose concentrations were measured in blood collected by venous bleeding from the tail vein before and 15 min, 30 min, 45 min, 60 min, 90 min, and 120 min after an intraperitoneal injection of 1.5 g/kg • body weight glucose using a Roche Diabetes Care glucometer (Roche, Germany).

An ITT was conducted on 17-week-old mice after a 6 h fast. Glucose concentrations were measured in blood collected by venous bleeding before and 15 min, 30 min, 45 min, and 60 min after the injection of insulin (Novolin, 30 R, 1.0 U/kg • body weight).

Plasma Parameters

The plasma biochemical parameters, including alanine transaminase (ALT), glucose, cholesterol, triglyceride (TG), high-density lipoprotein cholesterol (HDL-C), low-density lipoprotein cholesterol (LDL-C), and lactate dehydrogenase (LDH) levels, were determined by a 3100 Clinical Analyzer (Hitachi High-Technologies Corporation, Japan). The level of the plasma inflammation factor LPS was determined with an enzyme-linked immunosorbent assay (ELISA) kit (Thermo Fisher, USA) according to the operating instructions.

Histology and Oil Red O Staining

Tissues fixed in 4% paraformaldehyde were cut into 5 μm sections after being embedded in paraffin. Multiple sections were prepared and stained with hematoxylin and eosin (H&E) for general morphological observation.

Oil red O staining was performed as described by Ross et al.[32] Briefly, liver slices were washed with phosphate-buffered saline, fixed in 3.7% formaldehyde for 2 min, washed with H_2O, incubated with oil red O solution for 1 h at room temperature, and then washed with H_2O.

Immunohistochemistry and Immunofluorescence

Tissue sections for immunohistochemical testing were prepared on poly-L-lysine-pretreated

coverslips. Immunohistochemical staining was performed according to a standard protocol using antibodies against UCP1, TGR5, FXR1, SHP1 and SREBP-1c at a 1:500 dilution. The samples were incubated overnight in a humidified chamber at 4°C. Secondary antibodies for immunohistochemical staining were purchased from Invitrogen. All images were acquired on an Olympus BX51 system and processed using ImageJ software, version 1.8.0.
Immunostaining for mucin 2 was conducted using MUC2 primary antibody at a 1:1000 dilution. The samples were incubated overnight at 4°C. Secondary antibodies for immunofluorescence staining were diluted to 1:1500 and applied to the section for 2 h. Observations and analyses were performed with a Zeiss LSM 700 confocal microscope.

Western Blotting

Homogenized tissues were lysed in RIPA buffer containing protease and phosphatase inhibitors. The protein lysates were separated by SDS-PAGE. After electrophoresis, the proteins were transferred to a polyvinylidene difluoride membrane (Millipore), incubated with blocking buffer (5% fat-free milk) for 1 h at room temperature, and then blotted with the following antibodies overnight: anti-UCP1, anti-TGR5, anti-D2, anti-PGC-1α, anti-FXR1, anti-SHP1, anti-SREBP1 and anti-GAPDH. The membrane was incubated with HRP-conjugated secondary antibodies for 1 h at room temperature. The intensity values of the bands were quantified using ImageJ software.

Bomb Calorimetry

Fecal samples were collected from individual mice during the last week of the experiment and used for bomb calorimetry analysis. For bomb calorimetry analysis, the samples were weighed and oven-dried at 608°C for 48 h. The energy content of the feces was assessed with a Parr 6100 calorimeter using a 1109 semimicro bomb (Parr Instrument Co., Moline, Illinois, USA). The calorimeter energy equivalent factor was determined using benzoic acid standards, and each sample (100 mg) was analyzed in triplicate.

GM Analysis

The GM of the mice was analyzed as previously described.[30] Total genomic DNA was extracted from pooled samples using the CTAB/SDS method. The DNA concentration and purity were monitored on 1% agarose gels, and the DNA was diluted to 1 ng/μl in sterile water. 16S rRNA genes were amplified using a specific primer with a barcode. PCR was conducted in 30 μL reactions with 15 μL Phusion® High-Fidelity PCR Master Mix (New England Biolabs, USA), 0.2 μM forward and reverse primers, and approximately 10 ng template DNA. Thermal cycling consisted of an initial denaturation at 98 ℃ for 1 min, followed by 30 cycles of denaturation at 98 ℃ for 10 s, annealing at 50 ℃ for 30 s, and elongation at 72 ℃ for 30 s and, finally, 72 ℃ for 5 min. The PCR products were mixed in equal parts. The mixture was purified using a GeneJET Gel Extraction Kit (Thermo Scientific, USA). Sequencing libraries were generated using a TruSeq® DNA PCR-Free Sample Preparation Kit, following the manufacturer’s recommendations. Index codes were added. The library quality was assessed on a Qubit@ 2.0 Fluorometer (Thermo

Scientific) and an Agilent Bioanalyzer 2100 system. Finally, the library was sequenced on an Illumina HiSeq 2500, and 250 bp paired-end reads were generated.

Paired-end reads from the original DNA fragments were merged by using FLASH,[33] a high-speed and accurate analysis tool designed to merge paired-end reads when overlaps exist between read 1 and read 2. Paired-end reads were assigned to each sample according to their unique barcodes. The sequences were analyzed using the QIIME[34] (Quantitative Insights Into Microbial Ecology) software package. In-house Perl scripts were used to analyze alpha- (within samples) and beta- (among samples) diversity. First, the reads were filtered by QIIME quality filters. Then, pick_de_novo_otus.py was used to pick operational taxonomic units (OTUs) by creating an OTU table. Sequences with ≥ 97% similarity were assigned to the same OTUs. We picked a representative sequence for each OTU and used RDP classifier[35] to annotate taxonomic information for each representative sequence.

Statistical Analysis

All data reported in this paper are expressed as the means ± SEs. The data were evaluated by one-way ANOVA followed by Duncan's significant difference test. All statistics were analyzed by SPSS software, and all analyses were performed with GraphPad Prism 7.

REFERENCE

1. Jiang, T. T.; Shao, T. Y.; Ang, W. X. G.; Kinder, J. M.; Turner, L. H.; Pham, G.; Whitt, J.; Alenghat, T.; Way, S. S., Commensal Fungi Recapitulate the Protective Benefits of Intestinal Bacteria. *Cell host & microbe* **2017,** *22* (6), 809-816 e4.

2. Cho, I.; Yamanishi, S.; Cox, L.; Methe, B. A.; Zavadil, J.; Li, K.; Gao, Z.; Mahana, D.; Raju, K.; Teitler, I.; Li, H.; Alekseyenko, A. V.; Blaser, M. J., Antibiotics in early life alter the murine colonic microbiome and adiposity. *Nature* **2012,** *488* (7413), 621-626.

3. Zhang, Z.; Wu, X.; Cao, S.; Cromie, M.; Shen, Y.; Feng, Y.; Yang, H.; Li, L., Chlorogenic acid ameliorates experimental colitis by promoting growth of Akkermansia in mice. *Nutrients* **2017,** *9* (7), 677.

4. Jena, P. K.; Sheng, L.; Nagar, N.; Wu, C.; Barile, D.; Mills, D. A.; Wan, Y.-J. Y., The effect of synbiotics Bifidobacterium infantis and milk oligosaccharides on shaping gut microbiota community structure and NASH treatment. *Data in brief* **2018,** *19*, 1025-1029.

5. Christophersen, C.; Morrison, M.; Conlon, M., Overestimation of the abundance of sulfate-reducing bacteria in human feces by quantitative PCR targeting the Desulfovibrio 16S rRNA gene. *Appl. Environ. Microbiol.* **2011,** *77* (10), 3544-3546.

6. Ritchie, L. E.; Burke, K. F.; Garcia-Mazcorro, J. F.; Steiner, J. M.; Suchodolski, J. S., Characterization of fecal microbiota in cats using universal 16S rRNA gene and group-specific primers for Lactobacillus and Bifidobacterium spp. *Veterinary microbiology* **2010,** *144* (1-2), 140-146.

7. Tilg, H.; Cani, P. D.; Mayer, E. A., Gut microbiome and liver diseases. *Gut* **2016,** *65* (12), 2035-2044.

8. Zou, J.; Chassaing, B.; Singh, V.; Pellizzon, M.; Ricci, M.; Fythe, M. D.; Kumar, M. V.; Gewirtz, A. T., Fiber-mediated nourishment of gut microbiota protects against diet-induced obesity by restoring

IL-22-mediated colonic health. *Cell host & microbe* **2018,** *23* (1), 41-53. e4.
9. Moriguchi, T.; Yu, L.; Otsuki, A.; Ainoya, K.; Lim, K.-C.; Yamamoto, M.; Engel, J. D., Gata3 hypomorphic mutant mice rescued with a yeast artificial chromosome transgene suffer a glomerular mesangial cell defect. *Molecular and cellular biology* **2016,** *36* (17), 2272-2281.
10. Wen, C.-L.; Teng, C.-L.; Chiang, C.-H.; Chang, C.-C.; Hwang, W.-L.; Kuo, C.-L.; Hsu, S.-L., Methanol extract of Antrodia cinnamomea mycelia induces phenotypic and functional differentiation of HL60 into monocyte-like cells via an ERK/CEBP-β signaling pathway. *Phytomedicine* **2012,** *19* (5), 424-435.
11. Hyldahl, R. D.; Xin, L.; Hubal, M. J.; Moeckel-Cole, S.; Chipkin, S.; Clarkson, P. M., Activation of nuclear factor-κB following muscle eccentric contractions in humans is localized primarily to skeletal muscle-residing pericytes. *The FASEB Journal* **2011,** *25* (9), 2956-2966.
12. Zang, R.; Li, D.; Tang, I.-C.; Wang, J.; Yang, S.-T., Cell-based assays in high-throughput screening for drug discovery. *International Journal of Biotechnology for Wellness Industries* **2012,** *1* (1), 31-51.
13. Ho, R. H.; Leake, B. F.; Urquhart, B. L.; Gregor, J. C.; Dawson, P. A.; Kim, R. B., Functional characterization of genetic variants in the apical sodium‐dependent bile acid transporter (ASBT; SLC10A2). *Journal of gastroenterology and hepatology* **2011,** *26* (12), 1740-1748.
14. Howarth, D. L.; Law, S. H.; Law, J. M.; Mondon, J.; Kullman, S. W.; Hinton, D. E., Exposure to the synthetic FXR agonist GW4064 causes alterations in gene expression and sublethal hepatotoxicity in eleutheroembryo medaka (Oryzias latipes). *Toxicology and applied pharmacology* **2010,** *243* (1), 111-121.
15. Martinez-Lopez, N.; Tarabra, E.; Toledo, M.; Garcia-Macia, M.; Sahu, S.; Coletto, L.; Batista-Gonzalez, A.; Barzilai, N.; Pessin, J. E.; Schwartz, G. J., System-wide benefits of intermeal fasting by autophagy. *Cell metabolism* **2017,** *26* (6), 856-871. e5.
16. Bäckhed, F.; Ding, H.; Wang, T.; Hooper, L. V.; Koh, G. Y.; Nagy, A.; Semenkovich, C. F.; Gordon, J. I., The gut microbiota as an environmental factor that regulates fat storage. *Proceedings of the National Academy of Sciences* **2004,** *101* (44), 15718-15723.
17. Zhang, Y.-L.; Hernandez-Ono, A.; Siri, P.; Weisberg, S.; Conlon, D.; Graham, M. J.; Crooke, R. M.; Huang, L.-S.; Ginsberg, H. N., Aberrant hepatic expression of PPARγ2 stimulates hepatic lipogenesis in a mouse model of obesity, insulin resistance, dyslipidemia, and hepatic steatosis. *Journal of Biological Chemistry* **2006,** *281* (49), 37603-37615.
18. Bäckhed, F.; Manchester, J. K.; Semenkovich, C. F.; Gordon, J. I., Mechanisms underlying the resistance to diet-induced obesity in germ-free mice. *Proceedings of the National Academy of Sciences* **2007,** *104* (3), 979-984.
19. Tsuda, T.; Horio, F.; Uchida, K.; Aoki, H.; Osawa, T., Dietary cyanidin 3-O-β-D-glucoside-rich purple corn color prevents obesity and ameliorates hyperglycemia in mice. *The Journal of nutrition* **2003,** *133* (7), 2125-2130.
20. Kumar, D. P.; Rajagopal, S.; Mahavadi, S.; Mirshahi, F.; Grider, J. R.; Murthy, K. S.; Sanyal, A. J., Activation of transmembrane bile acid receptor TGR5 stimulates insulin secretion in pancreatic β cells. *Biochemical and biophysical research communications* **2012,** *427* (3), 600-605.
21. Marsili, A.; Ramadan, W.; Harney, J. W.; Mulcahey, M.; Castroneves, L. A.; Goemann, I. M.; Wajner, S. M.; Huang, S. A.; Zavacki, A. M.; Maia, A. L., Type 2 iodothyronine deiodinase levels are higher in slow-twitch than fast-twitch mouse skeletal muscle and are increased in hypothyroidism. *Endocrinology* **2010,** *151* (12), 5952-5960.
22. You, Y. L.; Han, X.; Guo, J. L.; Guo, Y.; Yin, M. W.; Liu, G. J.; Huang, W. D.; Zhan, J. C., Cyanidin-3-glucoside attenuates high-fat and high-fructose diet-induced obesity by promoting the

thermogenic capacity of brown adipose tissue. *Journal Of Functional Foods* **2018,** *41*, 62-71.
23. Albertoni, G.; Schor, N., Resveratrol plays important role in protective mechanisms in renal disease-mini-review. *Brazilian Journal of Nephrology* **2015,** *37* (1), 106-114.
24. Sevelda, F.; Mayr, L.; Kubista, B.; Lötsch, D.; van Schoonhoven, S.; Windhager, R.; Pirker, C.; Micksche, M.; Berger, W., EGFR is not a major driver for osteosarcoma cell growth in vitro but contributes to starvation and chemotherapy resistance. *Journal of Experimental & Clinical Cancer Research* **2015,** *34* (1), 134.
25. Elahi, S.; Ertelt, J. M.; Kinder, J. M.; Jiang, T. T.; Zhang, X.; Xin, L.; Chaturvedi, V.; Strong, B. S.; Qualls, J. E.; Steinbrecher, K. A.; Kalfa, T. A.; Shaaban, A. F.; Way, S. S., Immunosuppressive CD71+ erythroid cells compromise neonatal host defence against infection. *Nature* **2013,** *504* (7478), 158-62.
26. Li, Y.; Fromme, T.; Schweizer, S.; Schottl, T.; Klingenspor, M., Taking control over intracellular fatty acid levels is essential for the analysis of thermogenic function in cultured primary brown and brite/beige adipocytes. *EMBO Rep* **2014,** *15* (10), 1069-76.
27. You, Y.; Yuan, X.; Lee, H. J.; Huang, W.; Jin, W.; Zhan, J., Mulberry and mulberry wine extract increase the number of mitochondria during brown adipogenesis. *Food & function* **2014,** *6* (2), 401.
28. Goncalves, J.; Mendes, B.; Silva, C. L.; Camara, J. S., Development of a novel microextraction by packed sorbent-based approach followed by ultrahigh pressure liquid chromatography as a powerful technique for quantification phenolic constituents of biological interest in wines. *J Chromatogr A* **2012,** *1229*, 13-23.
29. Hagio, M.; Matsumoto, M.; Fukushima, M.; Hara, H.; Ishizuka, S., Improved analysis of bile acids in tissues and intestinal contents of rats using LC/ESI-MS. *J Lipid Res* **2009,** *50* (1), 173-80.
30. Guo, J. L.; Han, X.; Zhan, J. C.; You, Y. L.; Huang, W. D., Vanillin Alleviates High Fat Diet-Induced Obesity and Improves the Gut Microbiota Composition. *Frontiers In Microbiology* **2018,** *9:2733.*
31. Yuan, X.; Wei, G.; You, Y.; Huang, Y.; Lee, H. J.; Dong, M.; Lin, J.; Hu, T.; Zhang, H.; Zhang, C.; Zhou, H.; Ye, R.; Qi, X.; Zhai, B.; Huang, W.; Liu, S.; Xie, W.; Liu, Q.; Liu, X.; Cui, C.; Li, D.; Zhan, J.; Cheng, J.; Yuan, Z.; Jin, W., Rutin ameliorates obesity through brown fat activation. *FASEB J* **2017,** *31* (1), 333-345.
32. Ross, S. E.; Erickson, R. L.; Hemati, N.; Macdougald, O. A., Glycogen Synthase Kinase 3 Is an Insulin-Regulated C/EBPα Kinase. *Molecular & Cellular Biology* **1999,** *19* (12), 8433.
33. Magoč, T.; Salzberg, S. L., FLASH: fast length adjustment of short reads to improve genome assemblies. *Bioinformatics* **2011,** *27* (21), 2957-2963.
34. Caporaso, J. G.; Kuczynski, J.; Stombaugh, J.; Bittinger, K.; Bushman, F. D.; Costello, E. K.; Fierer, N.; Pena, A. G.; Goodrich, J. K.; Gordon, J. I., QIIME allows analysis of high-throughput community sequencing data. *Nature methods* **2010,** *7* (5), 335.
35. Wang, Q.; Garrity, G. M.; Tiedje, J. M.; Cole, J. R., Naïve Bayesian Classifier for Rapid Assignment of rRNA Sequences into the New Bacterial Taxonomy. *Applied & Environmental Microbiology* **2007,** *73* (16), 5261.
